# Immune checkpoints PVR and PVRL2 are prognostic markers in AML and their blockade represents a new therapeutic option

**DOI:** 10.1038/s41388-018-0288-y

**Published:** 2018-05-31

**Authors:** Hauke Stamm, Felix Klingler, Eva-Maria Grossjohann, Jana Muschhammer, Eik Vettorazzi, Michael Heuser, Ulrike Mock, Felicitas Thol, Gabi Vohwinkel, Emily Latuske, Carsten Bokemeyer, Roman Kischel, Cedric Dos Santos, Sabine Stienen, Matthias Friedrich, Michael Lutteropp, Dirk Nagorsen, Jasmin Wellbrock, Walter Fiedler

**Affiliations:** 1 Department of Oncology, Hematology and Bone Marrow Transplantation with Section Pneumology, Hubertus Wald University Cancer Center, University Medical Center Hamburg-Eppendorf, Hamburg, Germany; 2 Department of Medical Biometry and Epidemiology, University Medical Center Hamburg-Eppendorf, Hamburg, Germany; 3 Hematology, Hemostasis, Oncology and Stem Cell Transplantation, Hannover Medical School, Hannover, Germany; 4 Research Department Cell and Gene Therapy, Clinic for Stem Cell Transplantation, University Medical Center Hamburg-Eppendorf, Hamburg, Germany; 5 Amgen Research (Munich) GmbH, Munich, Germany; 6 Oncology Biomarkers and Early Clinical Development, Amgen, San Francisco, CA USA; 7 Global Clinical Development, Amgen, Thousand Oaks, CA USA

## Abstract

Immune checkpoints are promising targets in cancer therapy. Recently, poliovirus receptor (PVR) and poliovirus receptor-related 2 (PVRL2) have been identified as novel immune checkpoints. In this investigation we show that acute myeloid leukemia (AML) cell lines and AML patient samples highly express the T-cell immunoreceptor with Ig and ITIM domains (TIGIT) ligands PVR and PVRL2. Using two independent patient cohorts, we could demonstrate that high PVR and PVRL2 expression correlates with poor outcome in AML. We show for the first time that antibody blockade of PVR or PVRL2 on AML cell lines or primary AML cells or TIGIT blockade on immune cells increases the anti-leukemic effects mediated by PBMCs or purified CD3^+^ cells in vitro. The cytolytic activity of the BiTE® antibody construct AMG 330 against leukemic cells could be further enhanced by blockade of the TIGIT-PVR/PVRL2 axis. This increased immune reactivity is paralleled by augmented secretion of Granzyme B by immune cells. Employing CRISPR/Cas9-mediated knockout of PVR and PVRL2 in MV4-11 cells, the cytotoxic effects of antibody blockade could be recapitulated in vitro. In NSG mice reconstituted with human T cells and transplanted with either MV4-11 PVR/PVRL2 knockout or wildtype cells, prolonged survival was observed for the knockout cells. This survival benefit could be further extended by treating the mice with AMG 330. Therefore, targeting the TIGIT-PVR/PVRL2 axis with blocking antibodies might represent a promising future therapeutic option in AML.

## Introduction

Escape of neoplastic cells from immune destruction has recently been added to the list of hallmarks of cancer [1]. But, effector lymphocytes may acquire an exhausted phenotype during the course of the disease, preventing efficient tumor rejection [2, 3].

Inhibition of T-cell activation is accomplished by several receptor/ligand systems involved in checkpoint control of T-cell effector functions such as CTLA-4/CD80 and CD86 or PD-1/PD-L1 and PD-L2. Recently, therapeutic antibodies have been developed that inhibit these checkpoints resulting in reactivation of a cytotoxic phenotype. Clinical trials showed that CTLA-4 blocking antibodies ipilimumab or tremelimumab induced prolonged remissions in patients with malignant melanoma [4]. Antibodies against PD-1 such as pembrolizumab and nivolumab showed clinical activity in different tumor types including melanoma, Hodgkin's disease, renal, bladder and lung cancer [5, 6]. Currently, much effort is being directed toward the identification of novel immune checkpoint inhibitors [7].

A second class of immunotherapeutic agents are the bispecific T-cell engagers (BiTE®). BiTE® antibodies possess binding sites for CD3 on T cells and for tumor antigens, bringing neoplastic cells and T cells in close contact to induce their cytolytic action. Blinatumomab, a CD19/CD3 BiTE®, is the most advanced member in this class, and it is FDA and EMA approved for the treatment of acute lymphoblastic leukemia (ALL) [8]. For the treatment of acute myeloid leukemia (AML), AMG 330, a CD33/CD3 BiTE® antibody construct, has shown preclinical activity and is currently undergoing phase 1 clinical testing (NCT02520427) [9, 10]. Combining both approaches, tumor cell killing by T cells in the presence of BiTE® antibody constructs, as well as blockade of checkpoint molecules may result in enhanced therapeutic efficacy.

In the present investigation, we explored the therapeutic potential of inhibition of the novel immune regulators poliovirus receptor (PVR, CD155, Tage 4) and poliovirus receptor-related 2 (PVRL2, CD112, Nectin-2, PRR2), which bind to the CD28 family member T cell immunoreceptor with Ig and ITIM domains (TIGIT). TIGIT is a type I transmembrane protein with an Ig variable extracellular domain expressed on activated and memory T cells, regulatory T cells, as well as NK and NKT cells [11, 12]. Upon ligand interaction, TIGIT suppresses the immune response through its cytosolic immunoglobulin tail tyrosine (ITT)-like phosphorylation motif and immunoreceptor tyrosine-based inhibitory motif (ITIM) [13, 14]. PVR has been initially described as the poliovirus binding site and was linked to blood cells being an extraneural site for poliovirus replication [15, 16]. PVR is overexpressed by some tumor entities including melanoma, glioblastoma, colorectal and pancreatic carcinoma [17–20].

In our study, we analyzed the expression of TIGIT ligands PVR and PVRL2 on AML cell lines and patient samples and exploited the potential of this axis for the treatment of AML. For the first time, we show that blocking the TIGIT-PVR/PVRL2 axis augments T-cell mediated lysis of AML cells and additionally enhances the cytotoxic effects of the CD33/CD3 BiTE® antibody construct AMG 330.

## Results

### TIGIT ligands PVR and PVRL2 are highly expressed on AML cells

**Fig. 1:**
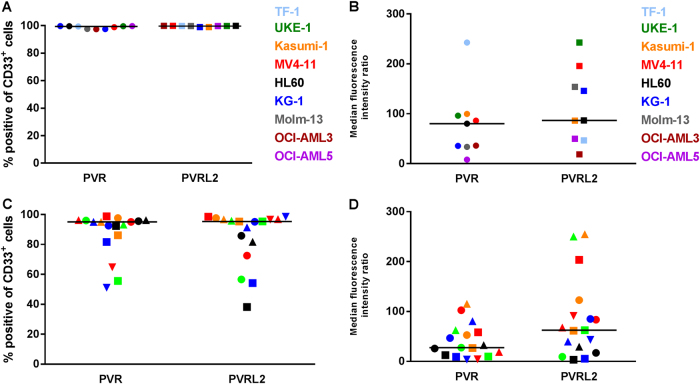
PVR and PVRL2 are highly expressed on AML cell lines and primary CD33^+^ AML blasts. PVR and PVRL2 protein expression, as depicted by the percentage of CD33^+^ cells as well as median fluorescence intensity as the measure of expression intensity on AML cell lines (*n* = 9; **a**, **b**) and CD33^+^ AML blasts from untreated patients (*n* = 17; **c**, **d**). Black dashes represent the median

The flow cytometric analysis revealed expression of PVR and PVRL2 on AML cell lines, all being CD33 positive (*n* = 9), and the majority of CD33^+^ AML blasts from untreated AML patients (*n* = 17; Fig. 1); see representative FACS plots in Supplemental Fig. 1). Furthermore, we could show that the majority of leukemia-initiating cells, defined as being CD34^+^/CD38^−^, expressed PVR and PVRL2 or at least one of the immune checkpoint ligands (mean expression value 77%, range 51–93%, *n* = 10). The expression of the corresponding receptor TIGIT was detected on 13.6 ± 3.7% of naïve CD3^+^ T cells from peripheral blood mononuclear cells of healthy donors (HD-PBMCs; *n* = 4, data not shown).

### Blockade of the TIGIT-PVR/PVRL2 axis significantly augments T-cell mediated lysis of AML cells alone or in combination with the BiTE® antibody construct AMG 330

**Fig. 2:**
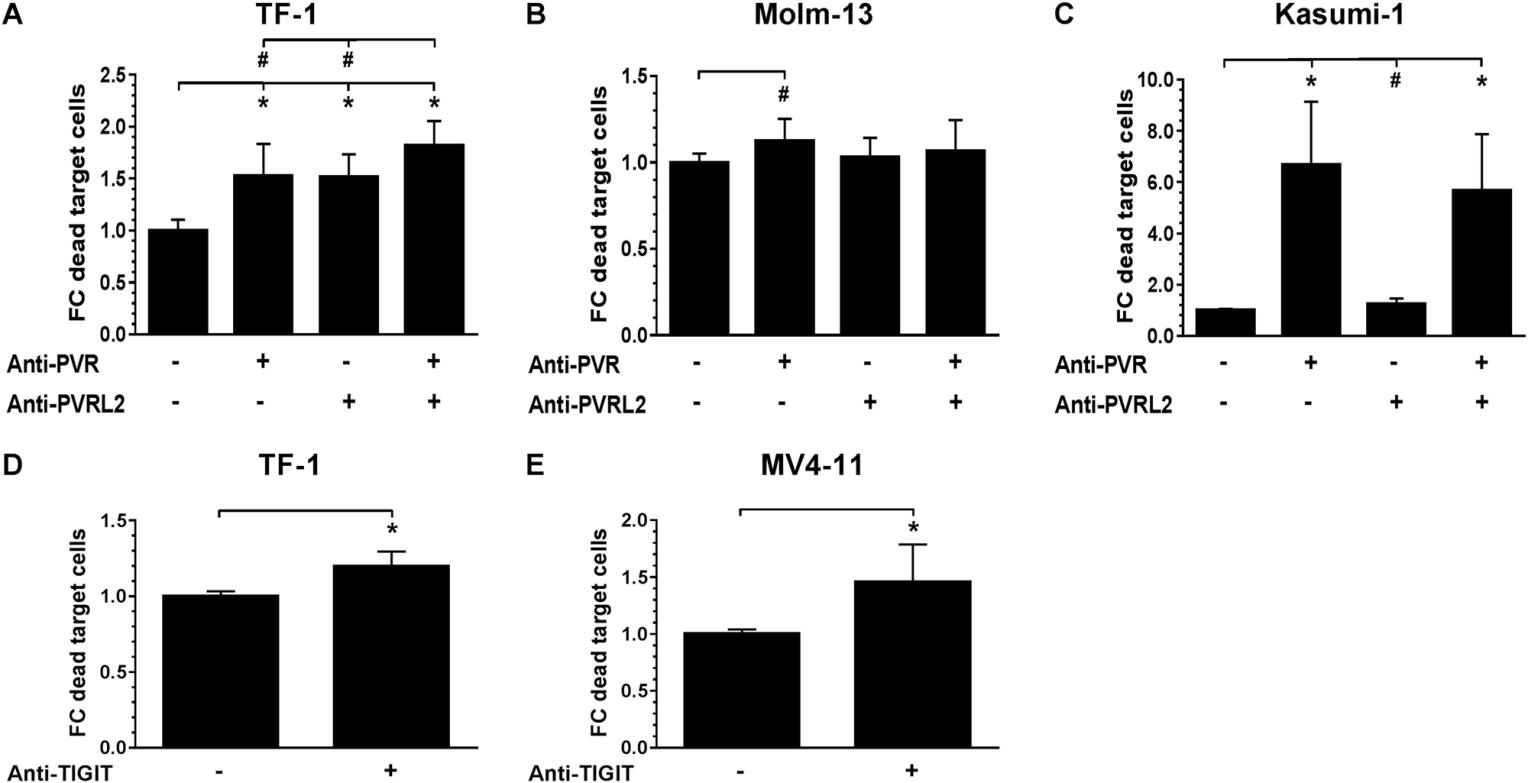
Blocking of the TIGIT-PVR/PVRL2 axis increases the lysis of AML cell lines. HD-PBMC-mediated lysis, with subject to the blocking of PVR and PVRLs on AML cell lines TF-1 (**a**, *n* = 3), Molm-13 (**b**, *n* = 6), Kasumi-1 (**c**, *n* = 3), was measured after 24 h. The effect of blocking the receptor TIGIT on effector cells was examined for the cell lines TF-1 (**d**, *n* = 5) and MV4-11 (**e**, *n* = 3). Results are depicted as the mean ± SD fold changes (FC) of dead target cells, relative to the control without blocking antibodies. Measurements were performed in technical triplicates and for statistical analysis Mann–Whitney *U*-tests were performed (#*p* ≤ 0.05; *p ≤ 0.001)

The therapeutic potential of blocking the novel immune checkpoint molecules PVR and PVRL2 on leukemic blasts was examined in in vitro cytotoxicity assays. The specific lysis of AML cells by HD-PBMCs in the presence or absence of blocking PVR/PVRL2 antibodies is depicted as the mean fold change of dead target cells normalized to the control without blocking antibodies for the cell lines TF-1, Molm-13 and Kasumi-1 (Fig. 2a–c). Since in MV4-11 cells only, a cytotoxic effect of the PVR and PVRL2 antibodies could be observed in the absence of HD-PBMCs (see Supplemental Fig. 2), we repeated the cytotoxicity assays using MV4-11 and TF-1 cells in the presence of a blocking anti-TIGIT antibody to replace the PVR/PVRL2 antibodies. Cell kill could be significantly augmented in both tested cell lines, indicating the importance of the TIGIT-PVR/PVRL2 axis as immune checkpoints (Fig. 2d, e).

**Fig. 3:**
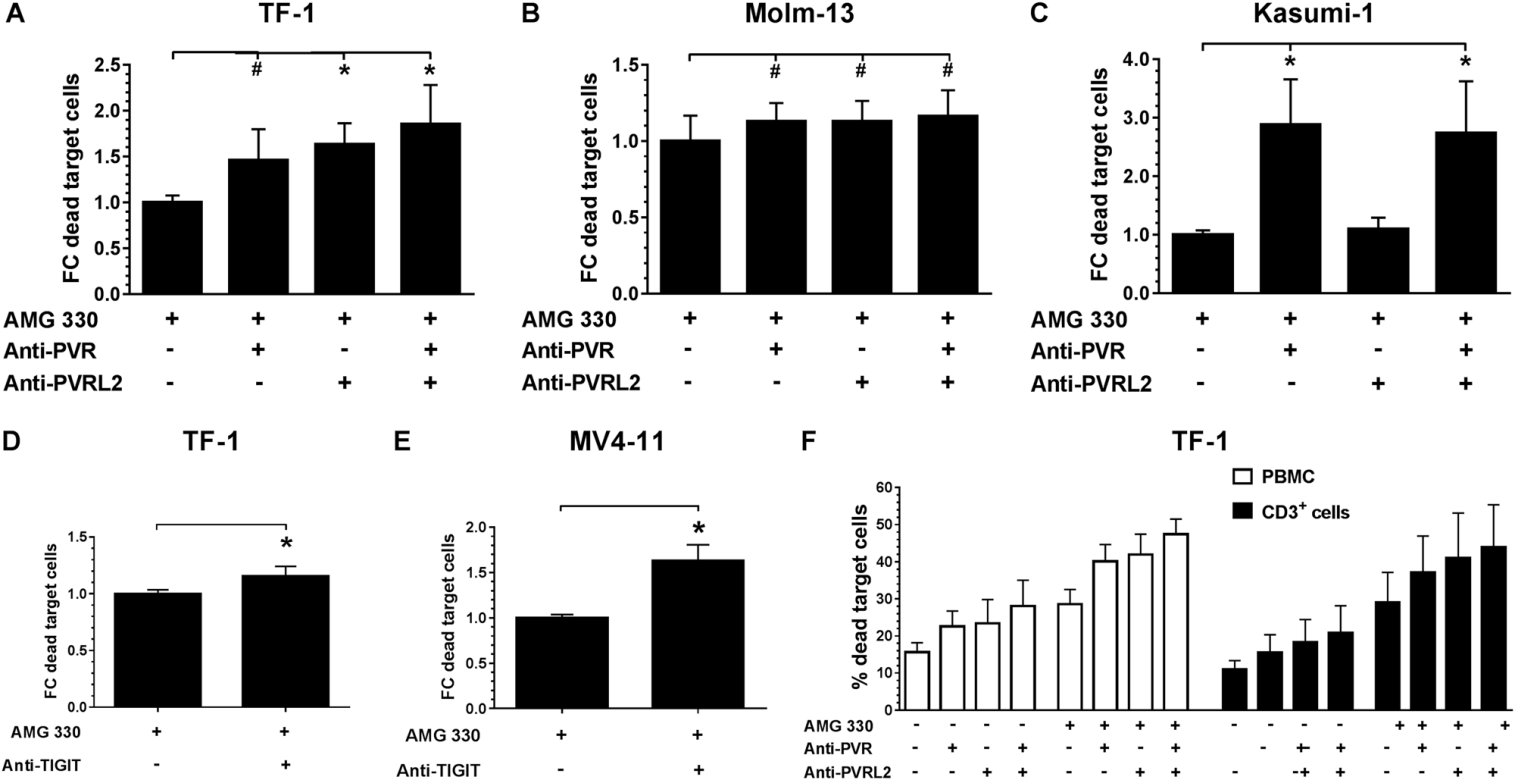
T-cell mediated lysis of the BiTE® antibody construct AMG 330 is significantly enhanced by additional administration of PVR and PVRL2 or TIGIT blocking antibodies. TF-1 (**a**, *n* = 3), Molm-13 (**b**, *n* = 6), Kasumi-1 (**c**, *n* = 6) cells were incubated with HD-PBMCs and AMG 330 in the presence or absence of blocking antibodies against PVR or PVRL2. Blocking the receptor TIGIT on immune cells showed similar results for the cell line TF-1 (**d**, *n* = 5) and MV4-11 (**e**, *n* = 3). Results are depicted as the mean ± SD fold change (FC) of dead target cells, relative to the control without blocking antibodies. Lysis is mediated via CD3^+^ cells, as comparing HD-PBMCs and purified CD3^+^ cells from the same donor showed comparable results using the cell line TF-1 (**f**, *n* = 2). Results are depicted as the mean ± SD of dead target cells of two independent experiments. Measurements were performed in technical triplicates, and for statistical analysis Mann–Whitney *U*-tests were performed (#*p* ≤ 0.05; *p ≤ 0.001)

**Fig. 4:**
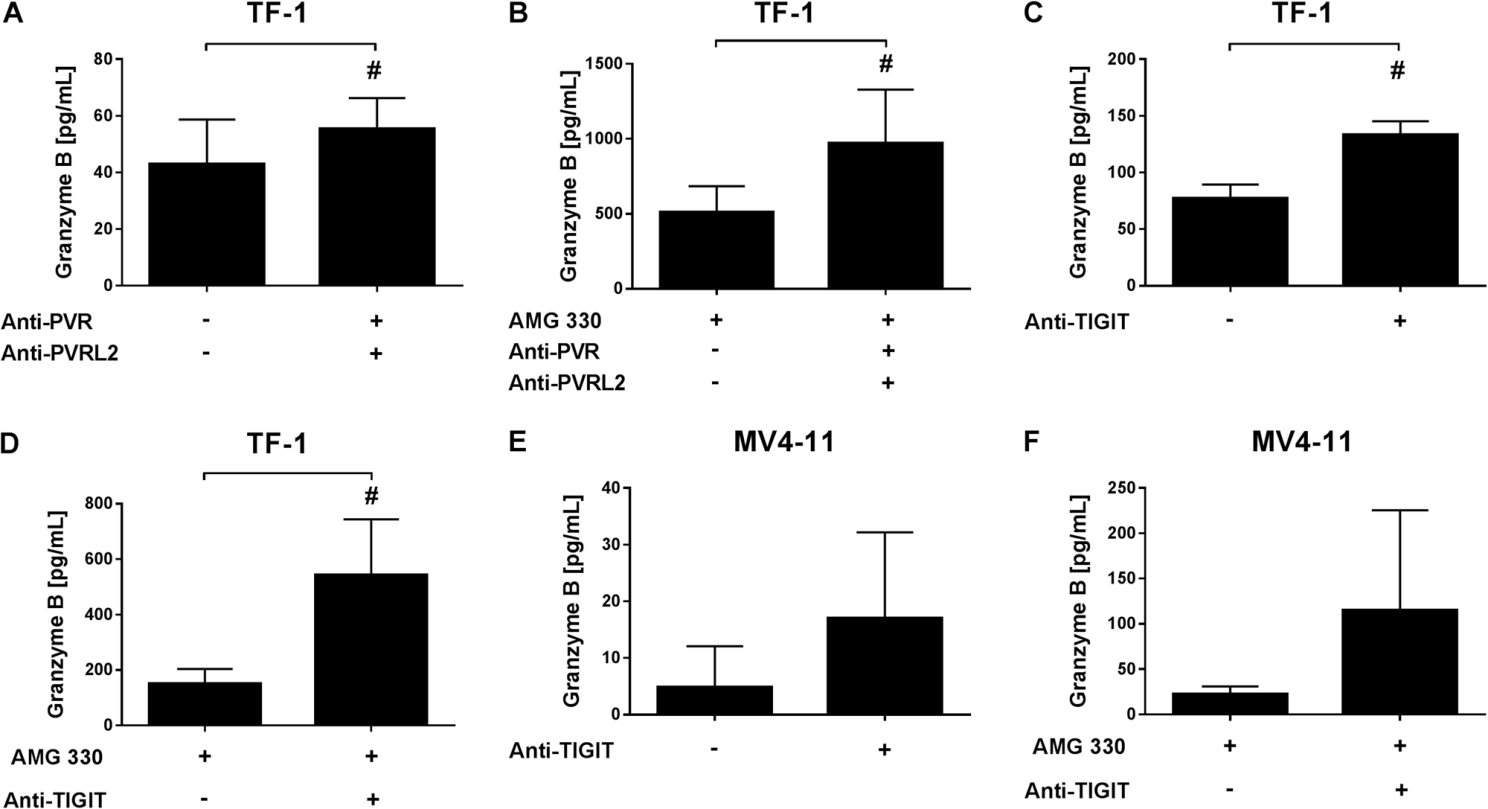
Blocking of the TIGIT-PVR/PVRL2 axis results in increased levels of Granzyme B secretion of immune cells. TF-1 (**a**–**d**) and MV4-11 (**e**, **f**) target cells were mixed with HD-PBMCs and incubated with blocking antibodies against PVR and PVRL2 or TIGIT in the presence or absence of the BiTE® antibody construct AMG 330. After 24 h, supernatants were harvested and human Granzyme B concentration was measured using ELISA. Results are depicted as the mean ± SD Granzyme B concentration of at least three independent experiments. For statistical analysis paired *t*-tests were performed (# *p* ≤ 0.05)

Further, we analyzed whether the T-cell mediated cytotoxicity exerted by AMG 330 could be enhanced by blocking PVR and PVRL2 or TIGIT. TF-1, Molm-13 or Kasumi-1 cells were incubated with HD-PBMCs or purified T cells for 24 h with or without 0.1 ng/mL AMG 330 in the presence or absence of PVR and PVRL2 blocking antibodies. Additionally, the killing of the cell lines MV4-11 and TF-1 was also investigated in the presence or absence of the anti-TIGIT antibody. As shown in Fig. 3a, the cytotoxic effect of AMG 330 was significantly increased by the administration of PVR or PVRL2 blocking antibodies using TF-1 cells (Fig. 3a). Significantly enhanced killing in comparison to the control could be detected after blocking PVR, PVRL2 or both antigens for the cell line Molm-13, although only moderate differences could be observed between all treatment arms (Fig. 3b). For Kasumi-1, only the PVR blocking antibody was able to induce a noteworthy enhancement of the cell lysis mediated by AMG 330 (Fig. 3c). The additional effect of blocking PVRL2 was neglectable in spite of high expression of PVRL2 on the cell line (Figs. 1, 3c). Employing the anti-TIGIT antibody, the effects of AMG 330 were augmented in a comparable manner (Fig. 3d, e). To exclude the effects of antibody dependent cellular cytotoxicity (ADCC), control experiments were performed. These included the use of an irrelevant antibody on c-kit (CD117) positive Kasumi-1 cells, and utilizing purified T cells as effectors (Supplemental Fig. S3 and S4 and Fig. 3f). Both controls emphasize the independence of ADCC in this context. To further investigate the T-cell mediated killing of target cells, we measured Granzyme B concentrations in the supernatants after incubation of TF-1 and MV4-11 with HD-PBMCs and antibodies against PVR, PVRL2, their combination or TIGIT. A significant increase in Granzyme B concentration was detected by the addition of blocking antibodies with or without AMG 330 (Fig. 4a–f, for a time course employing TF-1 cells see Supplemental Fig. S5).

### Blockade of PVR and PVRL2 significantly augments cytotoxicity against primary AML blasts

**Fig. 5:**
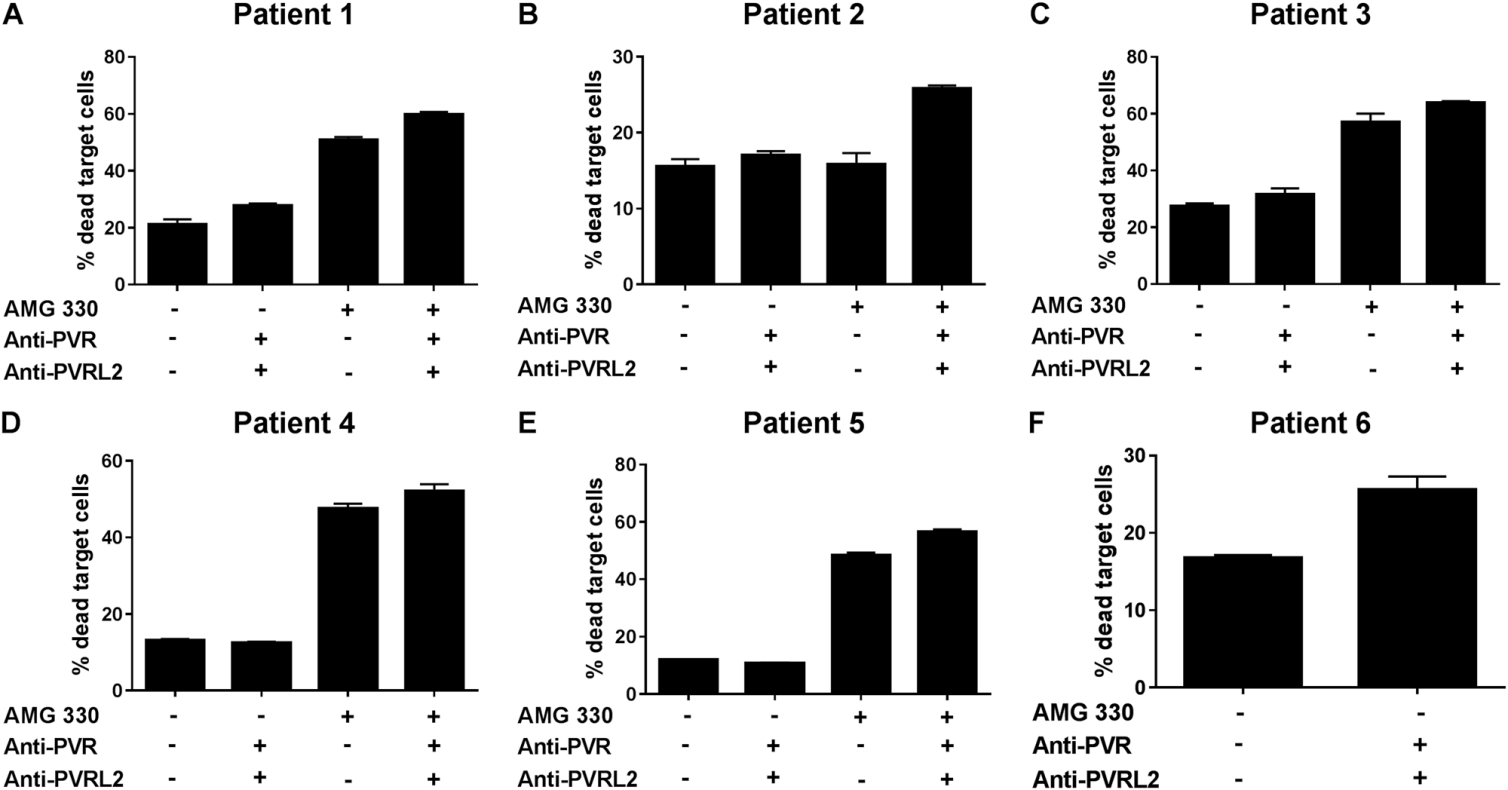
Combined blocking of PVR and PVRL2 on primary AML blasts increases the cytotoxic effects of HD-PBMCs. Mononuclear cells containing at least 75% blasts from bone marrow aspirates of ten different, newly diagnosed AML patients were stained with CMFDA (CellTracker™), mixed with HD-PBMCs as effector cells, and incubated for 72 h. Blocking of PVR and PVRL2 alone could increase the specific lysis of primary blasts in four (**a**, **b**, **c**, **f**) of the ten analyzed patients, and augment the anti-leukemic effect of the BiTE® antibody construct AMG 330 in five of nine patients (**a**–**e**). The sample of patient (**f**) was CD33 negative, and therefore excluded from the AMG 330 experiment. Results are depicted as the mean of technical triplicates ± SD of dead target cells

The effect of a PVR and PVRL2 blockade on the lysis of primary AML blasts from patient samples with high blast content was examined. Increased lysis of blasts due to the combined blocking of PVR and PVRL2 in four of the ten analyzed patient samples could be measured. The anti-leukemic effect of the BiTE® antibody construct AMG 330 could be enhanced in five out of nine CD33-expressing patient samples (Fig. 5a–f; see Supplemental Fig. S6 for non-responders).

### MV4-11 PVR and PVRL2 double-knockout cells recapitulate antibody effects

**Fig. 6:**
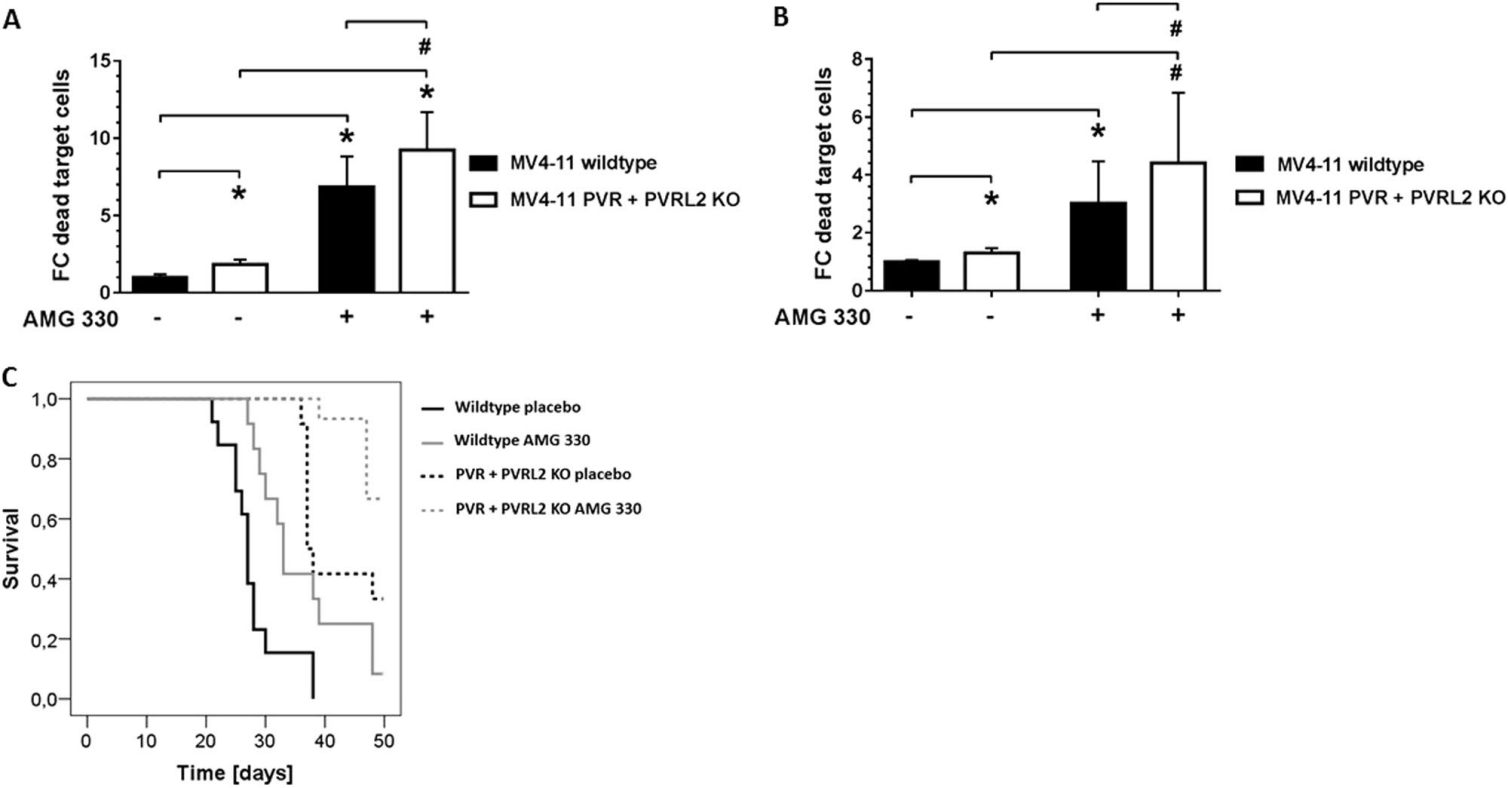
PVR and PVRL2 double-knockout cells recapitulate antibody effects in vitro and prolong the survival of NSG mice reconstituted with human T cells in vivo. By using CRISPR/Cas9, a polyclonal population of MV4-11 harboring double-knockout cells of PVR and PVRL2 was generated. Either MV4-11 wildtype or double-knockout cells were incubated with HD-PBMCs (**a**) or CD3^+^ cells (**b**) for 24 h without or with AMG 330. For statistical analysis, Mann–Whitney *U*-tests were performed (#*p* ≤ 0.05; *p ≤ 0.001, *n* = 3). **c** Immunodeficient NSG mice were transplanted with either MV4-11 wildtype (WT) cells or PVR- and PVRL2-double-knockout (KO) cells and reconstituted with human T cells. Treatment consisted of daily intraperitoneal application of either placebo (*n* = 13 for WT and *n* = 12 for KO) or 15 µg/kg AMG 330 (*n* = 12 for WT and *n* = 15 for KO). Log-rank tests were performed: WT placebo vs. KO placebo *p* < 0.001; WT AMG 330 vs. KO AMG 330 *p* < 0.001; WT placebo vs. WT AMG 330 *p* = 0.003; KO placebo vs. KO AMG 330 *p* = 0.027

To further strengthen our results, we generated PVR and PVRL2 double-knockouts for the cell line MV4-11 using CRISPR/Cas9. Cells harboring the double-knockout of PVR and PVRL2 on protein level were sorted by FACS (Supplemental Fig. S7). Furthermore, we analyzed the CRISPR/Cas9-induced genetic modifications resulting in the protein knockout in a number of single clones (Supplemental Fig. S8). The PVR and PVRL2 double-knockout cells were compared to their wildtype counterparts in cytotoxicity assays. In this setting, significantly enhanced killing of the double-knockout cells either by HD-PBMCs or purified T cells could be detected (Fig. 6a, b). This effect also resulted in higher secretion of Granzyme B during the kill of the double-knockout cells vs. the wildtype cells (data not shown). Addition of AMG 330 led again to a further significant increased cell lysis of the PVR and PVRL2 double-knockout cells compared to MV4-11 wildtype cells, indicating an augmented effect of combining both treatment approaches (Fig. 6a, b).

### Knockout of PVR and PVRL2 results in prolonged survival of NSG mice reconstituted with human T cells

To test the therapeutic impact of PVR and PVRL2 in vivo, in a blinded fashion, MV4-11 PVR and PVRL2 double-knockout cells or their wildtype counterpart were transplanted intravenously into immunodeficient NSG mice. Five days later, all mice were intraperitoneally injected with enriched and activated T cells from one blood donor. Mice received daily placebo or 15 µg/kg AMG 330. As shown in Fig. 6c, mice transplanted with the MV4-11 PVR and PVRL2 double-knockout cells had a significant survival benefit, compared to mice transplanted with MV4-11 wild-type cells, either in the placebo or AMG 330 arm (median survival of 37 vs. 27 days and 33 days vs. not reached during the 50 day observation period, respectively). Of note, MV4-11 wildtype cells had no growth advantage, compared to the MV4-11 double-knockout cells in vitro (Supplemental Fig. S9). Most importantly, in a preceding experiment with NSG mice not reconstituted with human T cells, no survival difference between the double-knockout and wildtype group was detectable, indicating no difference of AML engraftment and leukemia development in vivo (29 ± 3 vs. 29 ± 2 days, *n* = 4 and *n* = 3, respectively).

### Clinical significance: high expression of PVR and PVRL2 confers a negative prognosis to AML patients

To determine the expression of PVR and PVRL2, cDNA samples from 139 newly diagnosed AML patients enrolled into the AMLSG 07-04 study of the German-Austrian Study Group (NCT00151242) were analyzed by RT-qPCR (cohort A [21]). Patient characteristics are summarized in Supplemental Table S1. PVR and PVRL2 expression was found in 94% and 95% of AML patients, respectively. The level of gene expression and baseline clinical characteristics (age, karyotype and FLT3 mutation status) on event-free survival (EFS), relapse-free survival (RFS) and overall survival (OS) was investigated in a multivariate Cox proportional hazards model, implementing a stepwise removal of insignificant terms. Due to the low number of cases displaying a favorable karyotype, patients with intermediate and favorable karyotype were combined. High PVR expression as well as an unfavorable karyotype were identified as independent negative prognostic markers for EFS, RFS and OS, respectively (see Table 1 for hazard ratios and *p*-values). Due to a high correlation between PVR and PVRL2 (Pearson’s rho = 0.827, *p* < 0.001), PVRL2 was removed during the stepwise process. Nevertheless, if PVR was excluded from the multivariate cox regression, high PVRL2 expression represented a negative prognostic marker for RFS (*p* = 0.017), and had a borderline significant negative impact on OS (*p* = 0.087; see Table 1 for hazard ratios).

**Table 1:** Multivariate analysis of cohort A for event-free survival (EFS), relapse- free survival (RFS), and overall survival (OS)

Variable	Hazard ratio	95% CI	*p*-value
Including PVR			
EFS			
PVR expression	1.68	1.14–2.47	0.009
Karyotype	3.05	1.99–4.66	<0.001
RFS			
PVR expression	2.18	1.40–3.40	0.001
Karyotype	2.35	0.40–3.95	0.001
OS			
PVR expression	1.52	1.04–2.23	0.032
Karyotype	2.63	1.63–4.24	<0.001
Excluding PVR			
EFS			
PVRL2 expression	1.16	0.95–1.42	0.150
Karyotype	2.95	1.92–4.55	<0.001
RFS			
PVRL2 expression	1.30	1.05–1.61	0.017
Karyotype	2.19	1.29–3.72	0.004
OS			
PVRL2 expression	1.17	0.98–1.40	0.087
Karyotype	2.52	0.95–3.10	<0.001

*CI* confidence interval

As a validation cohort, the microarray-based gene expression data published by Verhaak et al. was used (cohort B [22]). The distribution of low vs. high PVR expressors was relatively equal in FAB type M2, M4 and M5, whereas it was significantly lower in AML M1 subgroup (Fisher’s exact test *p* = 0.01), while for PVRL2, no significant difference was observed in the various FAB subgroups (Fisher’s test *p* = 0.114; Supplemental Table S2). Due to the low number of 20 cases in total, FAB subtypes M0, M3, M6 and M7 were excluded from the subgroup analysis.

**Fig. 7:**
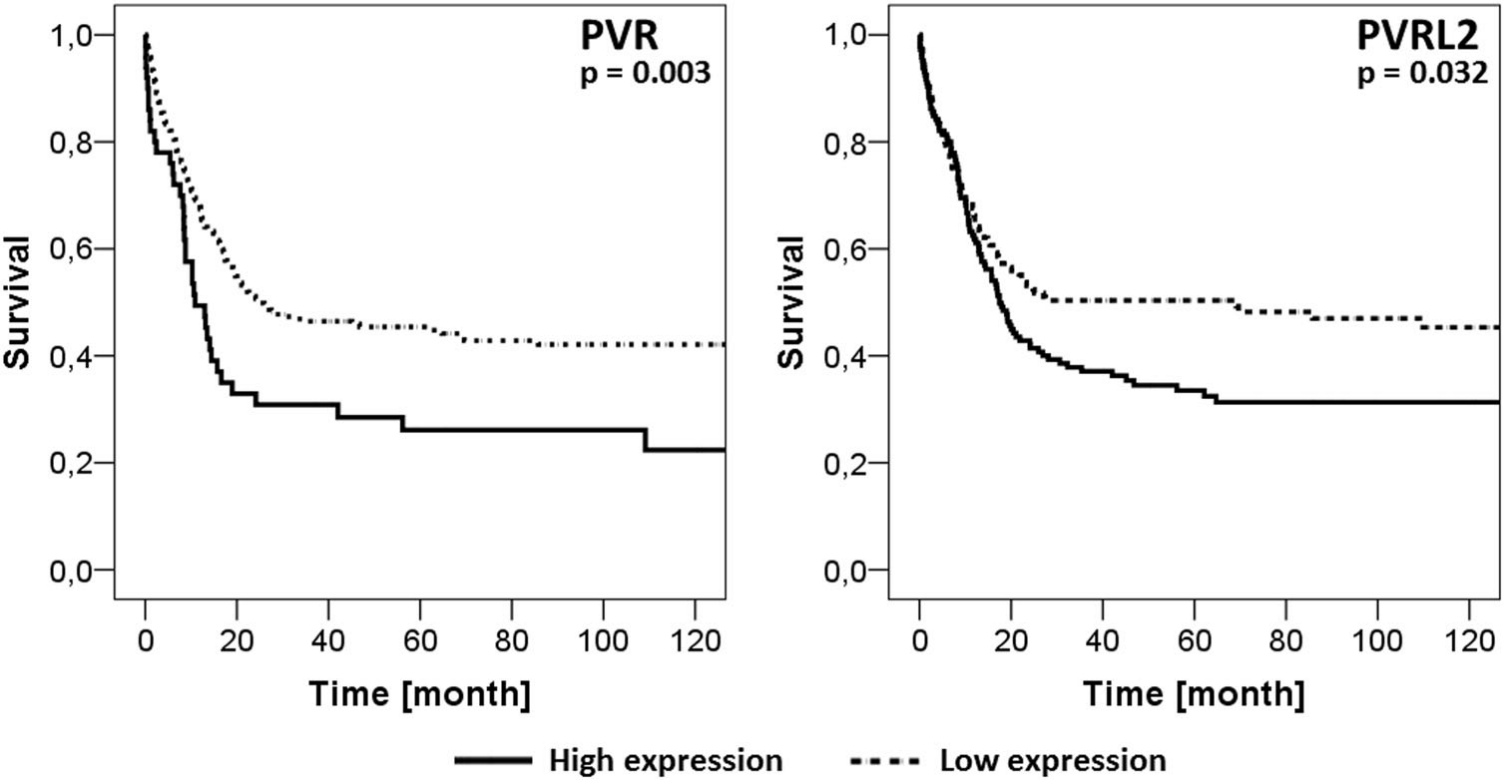
Impact of PVR and PVRL2 expression on clinical outcome. Microarray-based gene expression data of 290 AML patients (cohort B) were divided into low and high expressors and analyzed for OS. High expression of either PVR or PVRL2 correlated significantly with a shortened overall survival

In addition to PVR and PVRL2, we also analyzed the expression of CTLA-4 ligands CD80 and CD86 as well as PD-1 ligands PD-L1 and PD-L2. Clinical data of 290 AML patients were available enabling the analysis of the prognostic impact of gene expression of immune checkpoint molecules on OS (Supplemental Table S1 for patient characteristics). The patient cohort was divided into low vs. high expressors for the Kaplan–Meier survival analysis. The prognostic impact of PVR and PVRL2 could be confirmed, since PVR as well as PVRL2 high expressors displayed a significantly poorer OS, compared to the low expression groups (*p* = 0.003 and *p* = 0.032, respectively; Fig. 7). Interestingly, the CTLA-4 ligands CD80 and CD86 as well as the PD-1 ligand PD-L1 had no impact on the patient’s outcome, while PD-L2 expression was not detectable in the AML patients (Supplemental Fig. S10).

Furthermore, OS was analysed in a multivariate Cox proportional hazards model including the immune checkpoint ligands CD80, CD86, PD-L1, PVR, and PVRL2, and the baseline parameters age, karyotype and FLT3 mutational status. After a stepwise removal of insignificant terms, only high expression of PVR and a FLT3 mutation remained as independent prognostic survival markers (for PVR: HR 3.39 (95% CI 1.45–7.94), *p* = 0.005; for FLT3: HR 1.37 (95% CI 1.01–1.85), *p* = 0.045).

Of note, as no protein expression data was available for cohort A and B, we validated the correlation between the PVR and PVRL2 mRNA measured by RT-qPCR expression measured by flow cytometry in a set of pAML samples (*n* = 17; Pearson Rho 0.456, *p* = 0.066 for PVR and Pearson Rho, *p* = 0.009 for PVRL2).

## Discussion

Cancer immunotherapy targeting immune checkpoint receptor/ligand interactions is one of the most successful approaches in cancer therapy in the last years. Although blocking the signaling pathways of PD-1 or CTLA-4 showed clinical success, the majority of treated patients still responded poorly, emphasizing the need for additional strategies [23]. To our knowledge, with this study, we are the first to provide evidence on the therapeutic potential of blocking the TIGIT-PVR/PVRL2 axis for the treatment of AML. We show that AML cell lines as well as AML cells from untreated patients express PVR and PVRL2 to a high extent. The cytotoxic effects of effector cells were significantly enhanced in vitro by blocking these molecules on AML cell lines and primary blasts with antibodies against both ligands or against TIGIT on T cells. Moreover, by using this approach, the anti-leukemic effects of the BiTE® antibody construct AMG 330 were augmented. We demonstrate that these effects are independent of ADCC and specific to the blocking of PVR, PVRL2 or TIGIT. In line with these findings, MV4-11 cells lacking PVR and PVRL2 were more prone to cell lysis than MV4-11 wildtype cells in vitro, and prolonged the survival of NSG mice reconstituted with human T cells either with or without BiTE® antibody construct therapy. Furthermore, we show that the enhanced cytotoxicity of T cells is accompanied by increased release of Granzyme B. Most importantly, our analysis of two independent patient cohorts revealed that high PVR and PVRL2 expression was associated with a negative prognosis in AML implying immune evasion in these patients. Therefore, these novel immune regulators should be evaluated as targets for the treatment of AML.

BiTE® antibody constructs are investigated in a wide variety of solid and hematopoietic malignancies. The interaction of BiTE® antibody constructs with the target cancer cell leads to the formation of a T cell/target cell interaction, with the advantage of being independent of MHC class I interaction [24]. It has been shown that T-cell ligands influence the cytolytic activity of the BiTE® antibody construct AMG 330, where activating receptor/ligand interactions (e.g., CD28 with CD80/CD86) augmented and inhibitory ligand expression (e.g., PD-L1/PD-L2) impaired the cytotoxic activity of AMG 330 [25]. In agreement with these findings, the antibody blockade of the PD-1/PD-L1 axis reversed a T-cell-induced immune escape mechanism by enhancing the lysis of AML cells during AMG 330 treatment ex vivo [26]. Moreover, in a recent clinical case report, the possibility of resistance against the CD19/CD3 BiTE® antibody construct blinatumomab due to increased PD-L1 expression was described [27]. Considering the results of our study, we identified another important immune regulatory escape mechanism in AML, which serves as an attractive target for monotherapy as well as for combined administration with AMG 330.

In this report we show a high protein expression of the TIGIT ligands PVR and PVRL2 on AML cell lines and on most of the analysed CD33^+^ primary AML blasts. PVR and PVRL2 are physiologically expressed on hematopoietic cells and might be further upregulated by cancer cells as a mechanism of immune escape, whereas in AML, PD-L1 is not constitutively expressed, but upregulated by treatment with hypomethylating agents or during BiTE® antibody construct therapy [26, 28–30]. Two clinical studies have been recently presented in abstract form combining nivolumab with azacytidine or intensive chemotherapy, respectively. Here, additional immunotherapy targeting PD-1 resulted in only moderate improvement of the expected response rates [31, 32]. This favors the selection of PVR and PVRL2 as therapeutic targets over other immune checkpoints.

The exact mechanism by which PVR and PVRL2 exert their immunosuppressive function is still subject of debate. Both are also ligands to DNAX accessory molecule-1 (DNAM-1, CD226), which has immune activating properties for NK cells and T cells, promoting tumor cell lysis [33–35]. In analogy to the co-stimulatory and co-inhibitory receptor pair CD28 and CTLA-4, respectively, sharing the ligands CD80 and CD86, competitive binding of PVR to DNAM-1 or TIGIT has been proposed [36]. PVR and PVRL2 bind to the activating receptor DNAM-1 with low affinity and to the suppressing receptor TIGIT with high affinity. Additionally, CD96 has been identified on immune cells as a negative regulator of immune response competing with DNAM-1 for the common ligand PVR [37]. Nevertheless, there is accumulating evidence for the immunological importance of CD8^+^ effector function regulated by TIGIT. Johnston et al. showed that anti-viral and anti-tumor effector functions of CD8^+^ T cells are negatively regulated by TIGIT [38]. In our study, we describe that PVR and PVRL2 are important immune regulators for the immune surveillance of AML and that antibody blockade of these molecules augments anti-leukemic effects of cytotoxic T lymphocytes (CTL). Our findings are in line with a recent report in which melanoma cells expressing PVR control anti-melanoma CTL responses via the interaction between TIGIT and PVR in the effector phase by a suppression of cytokine release from melanoma-specific CTL [18].

Our results highlight the importance of the TIGIT-PVR/PVRL2 axis for the prognosis of AML patients under standard chemotherapy, implying that immune effects are also operational in this setting, since a high PVR and PVRL2 expression correlated with shorter overall survival in two independent patient cohorts. These results are strongly supported by a recent report stating that TIGIT contributes to functional T-cell impairment and that high TIGIT expression on T cells from AML patients correlates with poor clinical outcome [39]. Interestingly, a number of additional studies report a prognostic relevance for PVR in several tumor entities. Although no detectable immunohistological staining for PVR was found in normal tissue, PVR expression was elevated in some primary tumors including colorectal, prostate, renal and pancreatic carcinoma, melanoma as well as glioblastoma [17–20]. In support of our findings regarding the clinical significance for PVR and PVRL2 expression in AML, in pancreatic cancer patients, a high PVR expression also represented an independent prognostic factor for overall survival [20].

TIGIT is described as the dominant negative receptor with high affinity for PVR and low affinity for PVRL2 [11]. To our knowledge, all published studies concentrate on the impact of PVR on cancer outcome, whereas our findings highlight the equal importance of PVRL2 in this setting. This statement is espoused by the recent discovery of the novel inhibitory checkpoint receptor on human T cells, CD112R, which binds PVRL2. The authors show that disruption of this interaction enhances the human T-cell response [40]. As a third inhibitory receptor competing with DNAM-1 for ligands, the immune suppressive features of PVR and PVRL2 outweigh the immune stimulating properties. Moreover, in immune cells with exhausted phenotypes DNAM-1 is downregulated and might therefore not be able to display its role as immune activator [39, 41, 42]. Interestingly, the high expression of TIGIT on CD8^+^ T cells from AML patients observed by Kong and colleagues was inversely correlated to low DNAM-1 expression levels [39]. These findings might explain why immune stimulatory properties predominate upon blockade of PVR and PVRL2, as shown in our study.

Our preclinical findings indicate that the disruption of the TIGIT-PVR/PVRL2 axis might be of therapeutic value in patients. In this regard, a phase I and a phase I/II clinical study investigating the safety and tolerability of a TIGIT blocking antibody for the treatment of advanced and metastatic solid tumors is recruiting patients (NCT02913313 and NCT02794571). In addition, studies show that the inhibition of an immune response in cancer is often mediated via several receptors. In line with this, the combined administration of nivolumab and ipilimumab to patients with advanced melanoma resulted in a significantly prolonged RFS, compared to ipilimumab alone [43]. In multiple preclinical cancer models including AML, TIGIT expression was strongly associated with expression of other co-inhibitory molecules such as PD-1, Tim-3 and Lag-3, indicating the potential need for targeting multiple checkpoints in AML treatment [44, 45]. As shown in our study, the blocking of PVR and PVRL2 combined with other promising immunological approaches like BiTE® antibody construct therapies might be beneficial for cancer treatment [46, 47].

## Materials and methods

### Cell lines and cell culture

The AML cell lines in this study were recently verified using the multiplex human cell line authentication test (Multiplexion), and were tested for mycoplasma contamination using MycoAlert (Lonza). For culture conditions for AML cell lines, see Supplemental Methods.

### Patients and healthy donor samples

To analyze the clinical significance of PVR and PVRL2 expression in AML, two AML patient cohorts were studied. Cohort A comprised bone marrow and peripheral blood mononuclear cell (PBMCs) samples from 139 leukemic patients with newly diagnosed AML being treated within the AMLSG 07-04 study of the German-Austrian Study Group [21]. The second, independent patient cohort B comprised 290 AML patients of whom microarray-based gene expression data was published (GEO accession GSE6891 [22]).

Primary AML cells for in vitro experiments were isolated from bone marrow or PB using density gradient centrifugation. PBMCs were derived from healthy human cytomegalovirus seronegative, anonymous donors obtained from buffy coats kindly provided by the blood bank of the University Medical Center Hamburg-Eppendorf (Germany). T cells were isolated from PBMCs of a healthy donor using the Pan T Cell Isolation Kit and activated and enriched using the T Cell Activation/Expansion Kit (both Miltenyi Biotec) in RPMI 1640 + 10% FBS supplemented with 20 units/mL rIL2 (R&D Systems).

### Flow cytometry

Expression of PVR, PVRL2 and TIGIT was measured in flow cytometry using a BD FACSCalibur (BD Biosciences). A detailed protocol is provided in the Supplemental Methods section.

### Cytotoxicity assay

AML target cells were stained with 140 nM CellTracker™ Green CMFDA Dye (ThermoFisher) and mixed in a ratio of 1:6 with HD-PBMCs or CD3^+^ T cells at 1 × 10^6^ cells/mL. Two hundred microliter of cell suspension was plated in triplicates in 96-well plates in corresponding cell culture medium and incubated with or without 4 µg/mL blocking anti-PVR (clone D171, NeoMarkers), 25 µL/mL blocking anti-PVRL2 (clone L14 [35]), or 50 µg/mL blocking anti-TIGIT (Clone #A15153G, Biolegend) antibodies in the presence or absence of the BiTE® antibody construct AMG 330 (AMGEN Inc.) at 0.1 ng/mL. Assessment of specific lysis of the target cells was performed after 24 h of incubation by measuring the 7AAD (BD Biosciences) staining of gated CMFDA positive cells in flow cytometry. If not indicated differently, experiments were performed at least three times. To measure the lysis of AML blasts of de novo AML patients, the mononuclear cell fraction was isolated from bone marrow aspirates of newly diagnosed AML patients using density centrifugation. Only mononuclear cell fractions with more than 75% blasts were used. The mononuclear cells were stained with 205 nM CellTracker™ Green and the assay was performed as described above. Specific lysis was measured after an incubation time of 72 h.

### Granzyme B ELISA

For determining immune cell activation, Granzyme B concentration in supernatants of cytotoxicity assays was measured using the human Granzyme B DuoSet ELISA, according to manufacturer’s instructions.

### Generation of knockout cell lines using CRISPR/Cas9 technique

The PVR and PVRL2 double-knockout cell line MV4-11 was generated stepwise using CRISPR/Cas9 delivered by non-integrating lentiviral vectors (NILV). Details about the design, sequence of guide RNAs and the cloning protocol are provided in the Supplemental Methods section. Target cell lines were transduced with vector-containing supernatant and sorting of PVR/PVRL2 negative cells was performed by flow cytometry (FACS-Arialllu (BD Bioscience)). For analysis of CRISPR/Cas9-induced genomic alterations see Supplemental Methods.

### AML xenograft model

Either 2 × 10^5^ MV4-11 wildtype or PVR and PVRL2 double-knockout cells were injected intravenously into female 8-weeks-old NSG mice. Mice were reconstituted with 2 × 10^7^ enriched and activated T cells from a healthy donor 5 days later. Starting from day 8, mice were treated daily with PBS (placebo) or 15 µg/kg AMG 330 by intraperitoneal injection.

### Reverse transcription quantitative real-time PCR (RT-qPCR)

A detailed protocol of the RT-qPCR and data analysis for samples of cohort A is provided in the Supplemental Methods section. For primer sequences, see Supplemental Table S3.

### Statistics

All statistical analyses and graphical representations were done with SPSS 21 (SPSS) or GraphPad Prism® (GraphPad Software). In vitro analyses were compared using the Mann–Whitney *U*-test, a paired *t*-test, or Pearson correlation. To identify those gene expressions with independent significant predictive power, gene expressions were entered simultaneously into the same multivariable Cox model, and backwards selection was applied. Kaplan–Meier survival curves were calculated for different categories and compared with log-rank tests. See Supplemental Methods for more detailed information. *P*-values of ≤0.05 were considered as statistically significant.

### Study approval

The AMLSG 07-04 study of the German-Austrian Study Group (NCT00151242 [21]) was approved by the ethics committees of each study site and was conducted in accordance with the Austrian and German drug development regulations and the Declaration of Helsinki. Collection of samples from these patients was also approved by the Ethics Committee of the University of Ulm (148/10). Primary AML cells for in vitro experiments were obtained after patient’s informed consent and approval of the study by the local ethics committee (PV3469, Ethik Kommission der Ärztekammer Hamburg).

All animal experimental procedures in this study comply with the German Animal Welfare Act and the European Guideline EU 2010/63 and have been approved by the local authorities.

## Electronic supplementary material

Supplemental Methods

Supplemental Figure S1

Supplemental Figure S2

Supplemental Figure S3

Supplemental Figure S4

Supplemental Figure S5

Supplemental Figure S6

Supplemental Figure S7

Supplemental Figure S8

Supplemental Figure S9

Supplemental Figure S10

Supplemental Table S1

Supplemental Table S2

Supplemental Table S3
