# Supplementary material for: Immune checkpoints PVR and PVRL2 are prognostic markers in AML and their blockade represents a new therapeutic option
Source: Oncogene. 2018 May 31;37(39):5269–80. doi: 10.1038/s41388-018-0288-y (PMC6160395; doi:10.1038/s41388-018-0288-y)
Supplement: Supplementary file 1 — Supplemental Methods [file 41388_2018_288_MOESM1_ESM.docx]

Stamm *et al.,* “**Immune Checkpoints PVR and PVRL2 are Prognostic Markers in AML and Their Blockade Represents a New Therapeutic Option**”

**Supplemental Methods**

**Cell Culture**

For conventional suspension culture of AML cell lines, MV4-11 and Molm-13 cells were cultured at 37°C and 5% CO_2_ in RPMI 1640 medium (Life Technologies) supplemented with 10 % FBS Superior (Biochrome GmbH). TF-1 were cultured in RPMI 1640 supplemented with 10 % FBS and 2.5 ng/mL GM‑CSF (Peprotech). Kasumi-1 cells were cultured with RPMI 1640 supplemented with 20 % FBS. Primary AML blasts from de novo patients were cultured in ex vivo medium described elsewhere.(1)

**Flow cytometry**

For PVR and PVRL2 expression analysis, 0.2×10^6^ cells were labeled with APC conjugated anti-PVR (Clone SKII.4, BioLegend) or PE conjugated anti-PVRL2 (Clone TX31, BioLegend) antibodies with corresponding isotype controls. TIGIT or CD117 protein expression was assessed using an APC conjugated anti-TIGIT antibody (Clone #741182, R&D Systems) or anti-CD117 antibody (Clone #47233, R&D Systems) with corresponding isotype controls. Data was acquired on a BD FACSCalibur™ (BD Bioscience) and analyzed using FlowingSoftware (Version 2.5.1). The median fluorescence intensity (MFI) ratio (MFI target divided by MFI isotype control) was calculated as a measure of expression intensity. Expression of PVR and PVRL2 on leukemia inducing cells defined as CD34^+^/CD38^-^ was measured using a BV421 conjugated anti-CD38 (Clone HIT2, BioLegend) and a FITC conjugated anti-CD34 (Clone 561, BioLegend) antibody in combination with Zombie NIR Fixable Viability Kit (BioLegend). Experiments were performed on fresh and frozen samples with corresponding fluorescence minus one controls in each sample and measured on a BD FACSCanto™ II. Data was analyzed using FlowJo v10.0.7 (FlowJo, LLC).

**Generation of knockout cell lines using CRISPR/Cas9**

PVR and PVRL2 double knockout cell lines were generated stepwise using CRISPR/Cas9 delivered by non-integrating lentiviral vectors (NILV). Guide RNAs were designed using crispr.mit.edu and selected to be highly specific with quality score of 99 and 92 for PVR and PVRL2, respectively, with only allowing cross reactivity in other genes with at least four mismatches (guide PVR GATGTTCGGGTTGCGCGTAG; guide PVRL2 CGGCGATCTCGACGGCAGGA). Guide sequences were cloned into a lentiviral construct (U6-cgRNA-SFFV-Cas9-IRES-mCherry, derived from pX330 and LeGO-iC (www.addgene.org).(2) 3^rd^‑generation NILV were generated as described previously using the packaging plasmids pRSV-Rev, phCMV-VSV-G and pCMVD8.74D64V (www.addgene.org).(3) Target cell lines were transduced with vector-containing supernatant and sorting of PVR/PVRL2 negative cells was performed by flow cytometry using a FACS-Arialllu (BD Bioscience).

**Analysis of CRISPR/Cas9-induced genomic alterations**

To validate the knockout of the genes for *PVR* and *PVRL2* on genomic level, single cells were sorted and grown to monoclonal culture. Genomic DNA was isolated and the targeted sequence was amplified using primer “PVR forw subclone” and “PVR rev subclone” or “PVRL2 forw subclone” and “PVRL2 rev subclone” for *PVR* or *PVRL2*, respectively (supplemental table S3). Amplicons were subcloned into pJet1.2/blunt using the CloneJet PCR cloning kit (ThermoFisher Scientific) following the manufacturer’s protocol and sequenced using pJet1.2 forward sequencing primer (supplemental table S3). Results were analyzed using FinchTV Version 1.4.0 (Geospiza) and compared to MV4-11 wildtype sequence. Alterations to the wildtype sequence were incorporated into the reference cDNA sequence (NM_006505.3 for *PVR* and NM_001042724.1 for *PVRL2*) and effects on the protein sequence were examined using Translator (www.justbio.com).

**Analysis of proliferative capacity of CRISPR/Cas9-generated knockout cells**

The proliferative capacity of the polyclonal MV4-11 PVR and PVRL2 double knockout cells used for *in vitro* cytotoxicity assays and the *in vivo* AML xenograft mouse model was compared to the MV4-11 wildtype counterpart in a four day kinetic cell proliferation assay. Respective cells were grown under exact equal culture conditions for at least one week in advance. At the experiment start, 5 mL with 1×10^5^ cells/mL were plated in triplicates in a 6 well plate and the concentration of viable cells was counted in triplicates at day 2 and day 4, respectively, using a Vi-Cell™XR automatic cell counter (Beckman Coulter).

**Reverse transcription quantitative real-time PCR (RT-qPCR)**

For samples of cohort A, total RNA of 1×10^7^ cells was extracted using the TRizol method (Invitrogen) and reversely transcribed into cDNA using MuLV-RT (Invitrogen). RT-qPCR analysis was performed on the LightCycler® 96 (Roche) using SYBR® Ex Taq™ II (Takara Bio). Relative amounts of target cDNA were calculated relative to GAPDH expression as described elsewhere.(4) PCR efficiencies were calculated using standard curves obtained from log dilutions of plasmids containing the corresponding gene sequences. For primer sequences see supplemental table S3.

**Statistical analysis of patient cohorts**

Overall survival (OS) was defined as time from study inclusion to death. Relapse-free survival (RFS) was defined as time from achieving a complete remission (CR) after the first therapy to relapse or death from any cause, while event-free survival (EFS) was defined as time from study inclusion to any predefined event (first therapy failure, relapse or death). To identify those gene expressions with independent significant predictive power, gene expressions were entered simultaneously into the same multivariable Cox model and a backwards selection was applied. For Kaplan-Meier survival analysis, Classification-and-Regression-Tree (CART) analysis, a method to subdivide the study population into subgroups of different risks, was used to derive cut-off values on gene expression data for overall mortality risks.(5) If no convenient cut-off level could be determined, the median of the gene expression values was used to categorize the AML patient cohort into high vs. low expression groups. Kaplan-Meier survival curves were calculated for different categories and compared with log-rank tests. P‑values of ≤0.05 were considered significant.

**Supplementary References**

1. Pabst C, Krosl J, Fares I, Boucher G, Ruel R, Marinier A, et al. Identification of small molecules that support human leukemia stem cell activity ex vivo. Nat Methods. 2014 Apr;11(4):436–42.

2. Cong L, Ran FA, Cox D, Lin S, Barretto R, Habib N, et al. Multiplex genome engineering using CRISPR/Cas systems. Science. 2013 Feb 15;339(6121):819–23.

3. Yáñez-Muñoz RJ, Balaggan KS, MacNeil A, Howe SJ, Schmidt M, Smith AJ, et al. Effective gene therapy with nonintegrating lentiviral vectors. Nat Med. 2006 Mar 19;12(3):348–53.

4. Pfaffl MW. A new mathematical model for relative quantification in real-time RT-PCR. Nucleic Acids Res. 2001 May 1;29(9):e45.

5. Hothorn T, Hornik K, Zeileis A. Unbiased Recursive Partitioning: A Conditional Inference Framework. J Comput Graph Stat. Taylor & Francis; 2006 Sep 1;15(3):651–74.
