# Supplementary material for: Immune checkpoints PVR and PVRL2 are prognostic markers in AML and their blockade represents a new therapeutic option
Source: Oncogene. 2018 May 31;37(39):5269–80. doi: 10.1038/s41388-018-0288-y (PMC6160395; doi:10.1038/s41388-018-0288-y)
Supplement: Supplementary file 2 — Supplemental Figure S1 [file 41388_2018_288_MOESM2_ESM.docx]

Stamm *et al.,* “**Immune Checkpoints PVR and PVRL2 are Prognostic Markers in AML and Their Blockade Represents a New Therapeutic Option**”


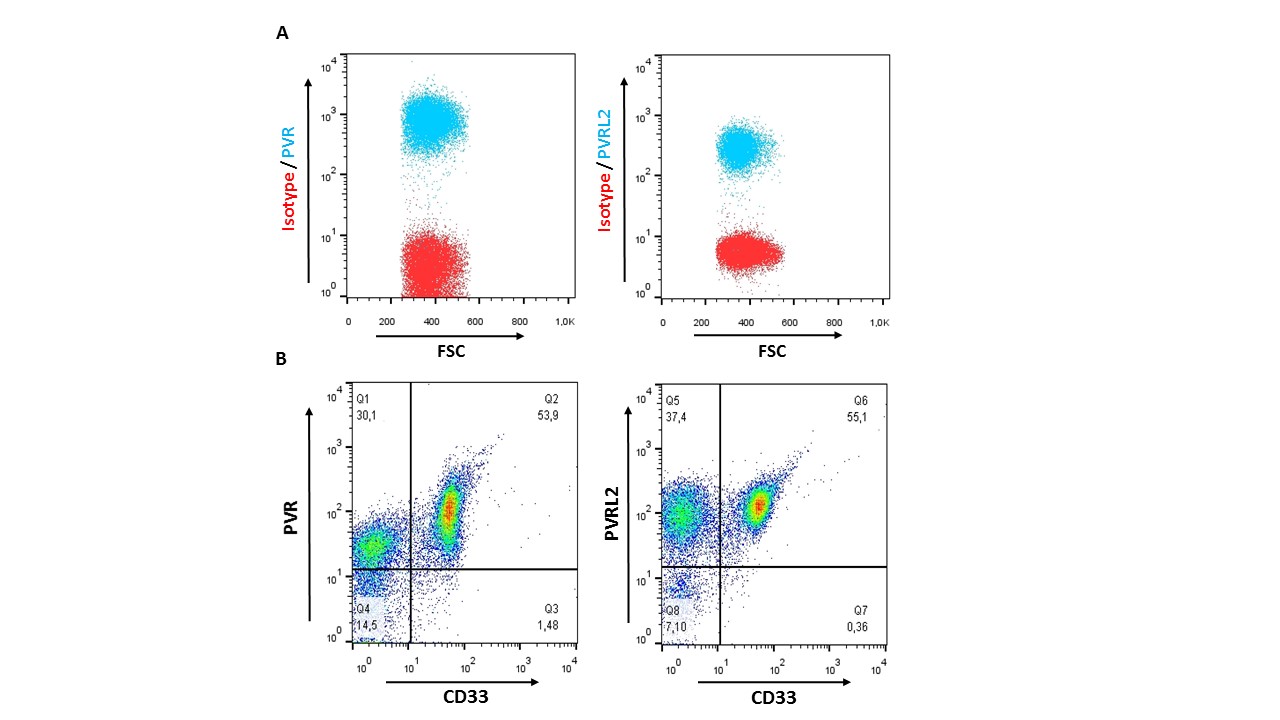


**Supplemental Figure S1. Representative FACS plots of the AML cell line TF-1 (A) and a primary AML sample (B).** PVR, PVRL2 and CD33 expression was measured by flow cytometry.
