# Supplementary material for: Immune checkpoints PVR and PVRL2 are prognostic markers in AML and their blockade represents a new therapeutic option
Source: Oncogene. 2018 May 31;37(39):5269–80. doi: 10.1038/s41388-018-0288-y (PMC6160395; doi:10.1038/s41388-018-0288-y)
Supplement: Supplementary file 3 — Supplemental Figure S2 [file 41388_2018_288_MOESM3_ESM.docx]

Stamm *et al.,* “**Immune Checkpoints PVR and PVRL2 are Prognostic Markers in AML and Their Blockade Represents a New Therapeutic Option**”


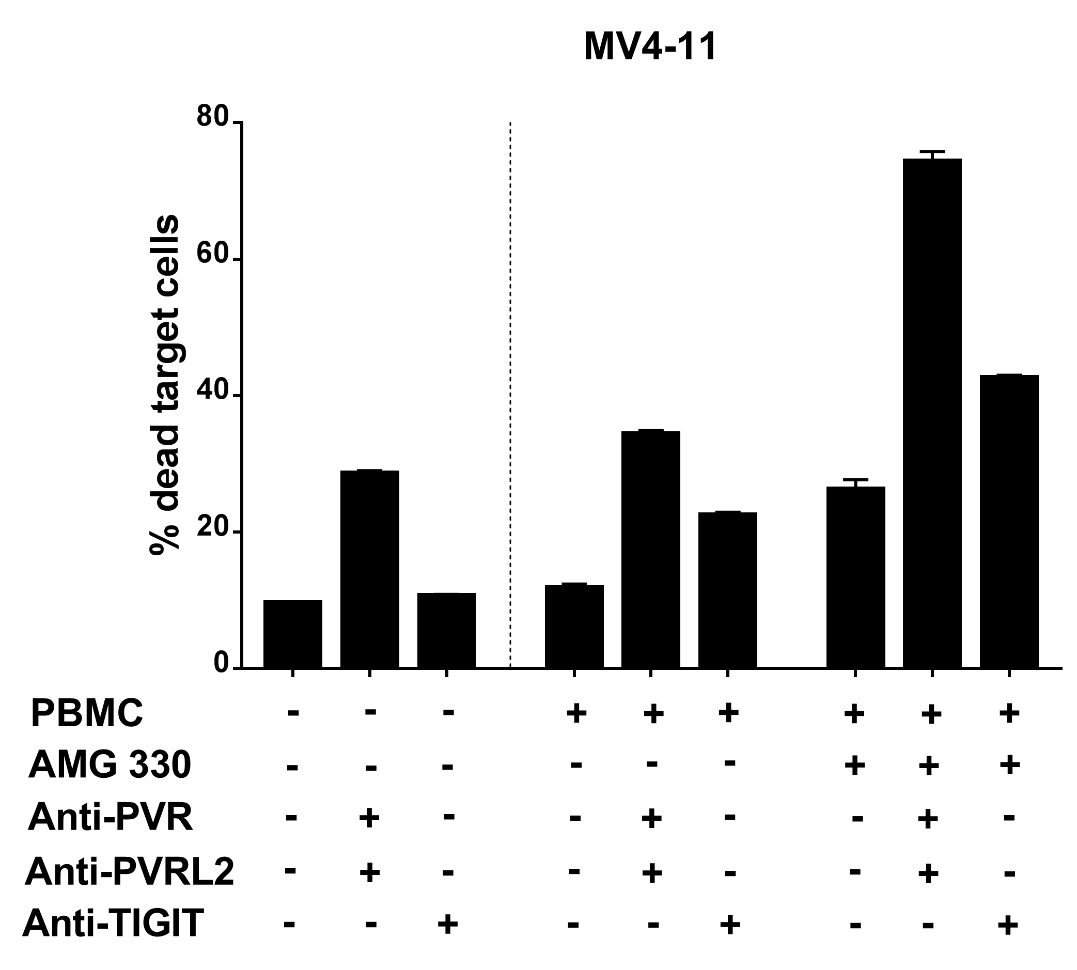


**Supplemental Figure S2. Representative killing assay for the cell line MV4-11.** MV4-11 cells were incubated without or with HD-PBMCs and AMG 330 in the presence of absence of blocking antibodies against PVR, PVRL2 or TIGIT. Results are depicted as the mean ± SD of dead target cells and measurements were performed in technical triplicate.
