# Supplementary material for: Immune checkpoints PVR and PVRL2 are prognostic markers in AML and their blockade represents a new therapeutic option
Source: Oncogene. 2018 May 31;37(39):5269–80. doi: 10.1038/s41388-018-0288-y (PMC6160395; doi:10.1038/s41388-018-0288-y)
Supplement: Supplementary file 4 — Supplemental Figure S3 [file 41388_2018_288_MOESM4_ESM.docx]

Stamm *et al.,* “**Immune Checkpoints PVR and PVRL2 are Prognostic Markers in AML and Their Blockade Represents a New Therapeutic Option**”

**
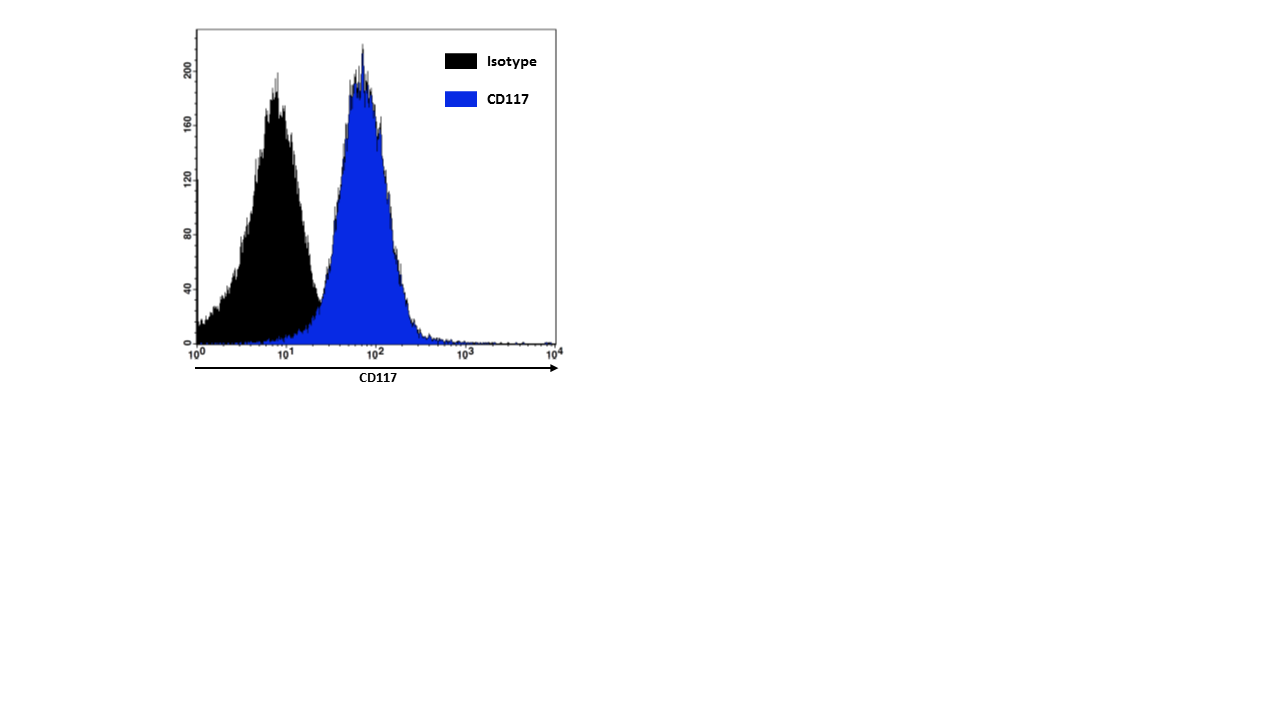
**

**Supplemental Figure S3. FACS analysis for CD117 protein expression on Kasumi-1.** CD117 expression was measured by flow cytometry.
