# Supplementary material for: Immune checkpoints PVR and PVRL2 are prognostic markers in AML and their blockade represents a new therapeutic option
Source: Oncogene. 2018 May 31;37(39):5269–80. doi: 10.1038/s41388-018-0288-y (PMC6160395; doi:10.1038/s41388-018-0288-y)
Supplement: Supplementary file 5 — Supplemental Figure S4 [file 41388_2018_288_MOESM5_ESM.docx]

Stamm *et al.,* “**Immune Checkpoints PVR and PVRL2 are Prognostic Markers in AML and Their Blockade Represents a New Therapeutic Option**”

**
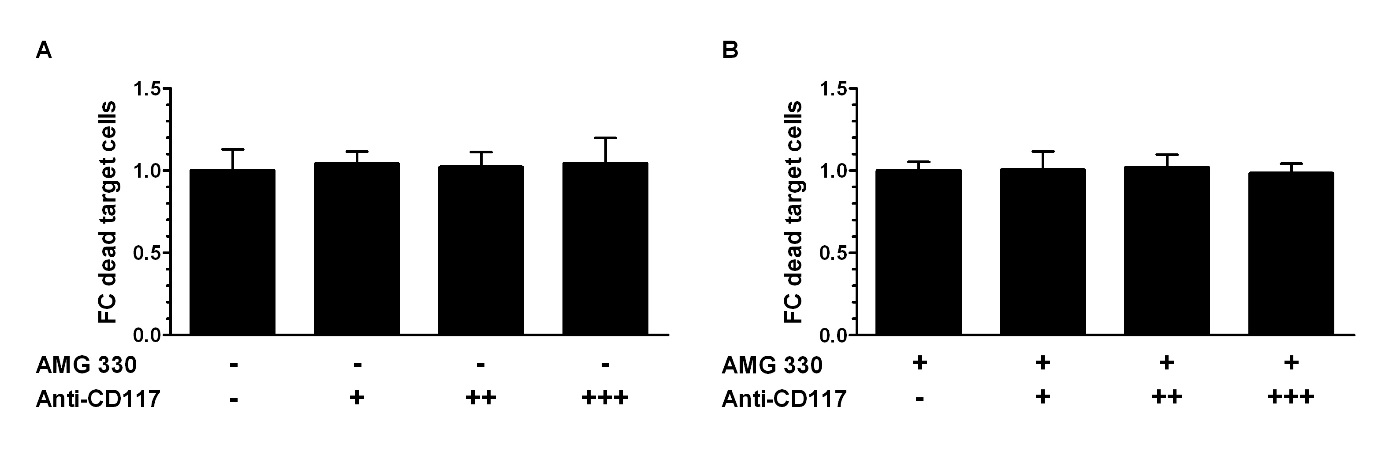
**

**Supplemental Figure S4. The increase of cell lysis by blocking PVR and PVRL2 is specific and not mediated via ADCC.** Kasumi-1 cells were incubated with PBMCs and escalating doses of an antibody targeting CD117 in the presence or absence of AMG 330 (n=2, + 2µg/mL, ++ 10 µg/mL, +++ 50 µg/mL). Results are depicted as the mean ± SD of fold changes (FC) of dead target cells normalized to the control.
