# Supplementary material for: Immune checkpoints PVR and PVRL2 are prognostic markers in AML and their blockade represents a new therapeutic option
Source: Oncogene. 2018 May 31;37(39):5269–80. doi: 10.1038/s41388-018-0288-y (PMC6160395; doi:10.1038/s41388-018-0288-y)
Supplement: Supplementary file 6 — Supplemental Figure S5 [file 41388_2018_288_MOESM6_ESM.docx]

Stamm *et al.,* “**Immune Checkpoints PVR and PVRL2 are Prognostic Markers in AML and Their Blockade Represents a New Therapeutic Option**”

**
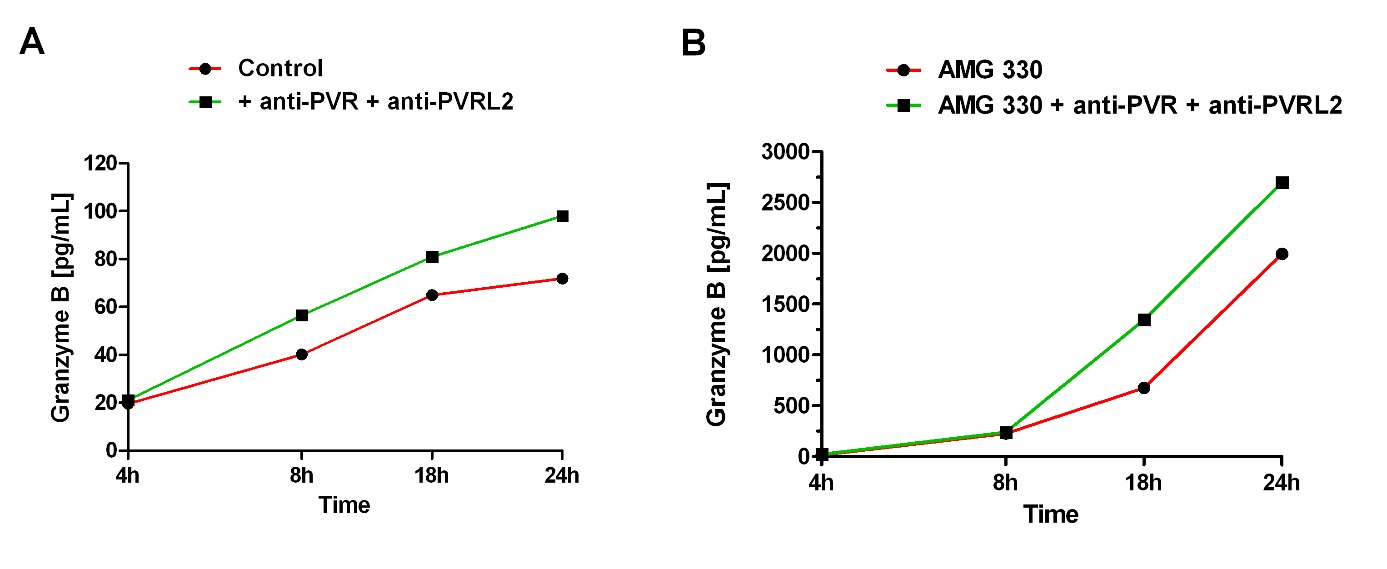
**

**Supplemental Figure S5. Time course of immune-cell mediated granzyme B release.** TF-1 cells were incubated with HD-PBMCs and antibodies against PVR and PVRL2 alone (A) or in combination with AMG 330 (B). The culture supernatants were harvested at different time points and granzyme B release was measured using the human granzyme B DuoSet ELISA kit in technical duplicates (R&D Systems).
