# Supplementary material for: Immune checkpoints PVR and PVRL2 are prognostic markers in AML and their blockade represents a new therapeutic option
Source: Oncogene. 2018 May 31;37(39):5269–80. doi: 10.1038/s41388-018-0288-y (PMC6160395; doi:10.1038/s41388-018-0288-y)
Supplement: Supplementary file 7 — Supplemental Figure S6 [file 41388_2018_288_MOESM7_ESM.docx]

Stamm *et al.,* “**Immune Checkpoints PVR and PVRL2 are Prognostic Markers in AML and Their Blockade Represents a New Therapeutic Option**”


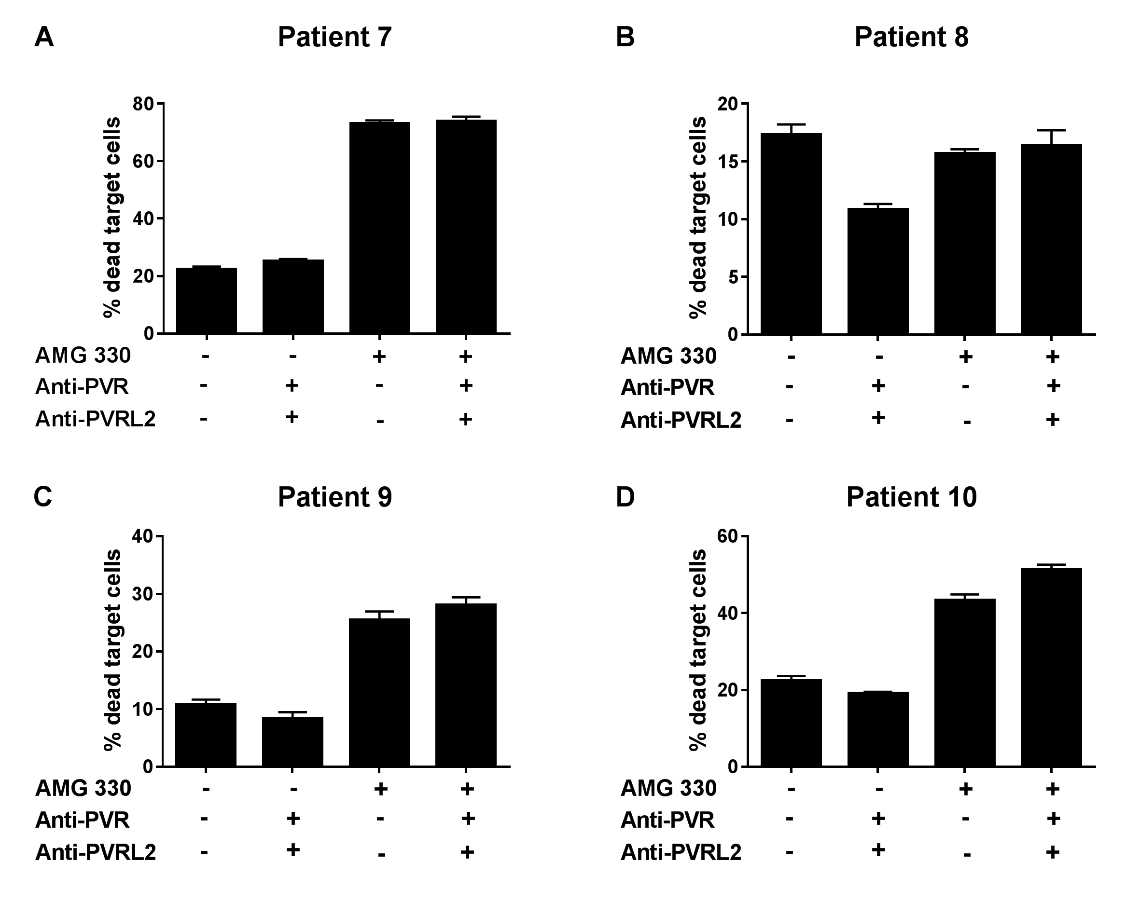


**Supplemental Figure S6. Demonstration of patient cases not or only partially responding to immune checkpoint blockade.** Mononuclear cells containing at least 75 % blasts from bone marrow aspirates of newly diagnosed AML patients were stained with CMFDA (CellTracker™), mixed with HD-PBMCs as effector cells and incubated for 72 h in the presence or absence of blocking antibodies against PVR and PVRL2.
