# Supplementary material for: Immune checkpoints PVR and PVRL2 are prognostic markers in AML and their blockade represents a new therapeutic option
Source: Oncogene. 2018 May 31;37(39):5269–80. doi: 10.1038/s41388-018-0288-y (PMC6160395; doi:10.1038/s41388-018-0288-y)
Supplement: Supplementary file 8 — Supplemental Figure S7 [file 41388_2018_288_MOESM8_ESM.docx]

Stamm *et al.,* “**Immune Checkpoints PVR and PVRL2 are Prognostic Markers in AML and Their Blockade Represents a New Therapeutic Option**”

**
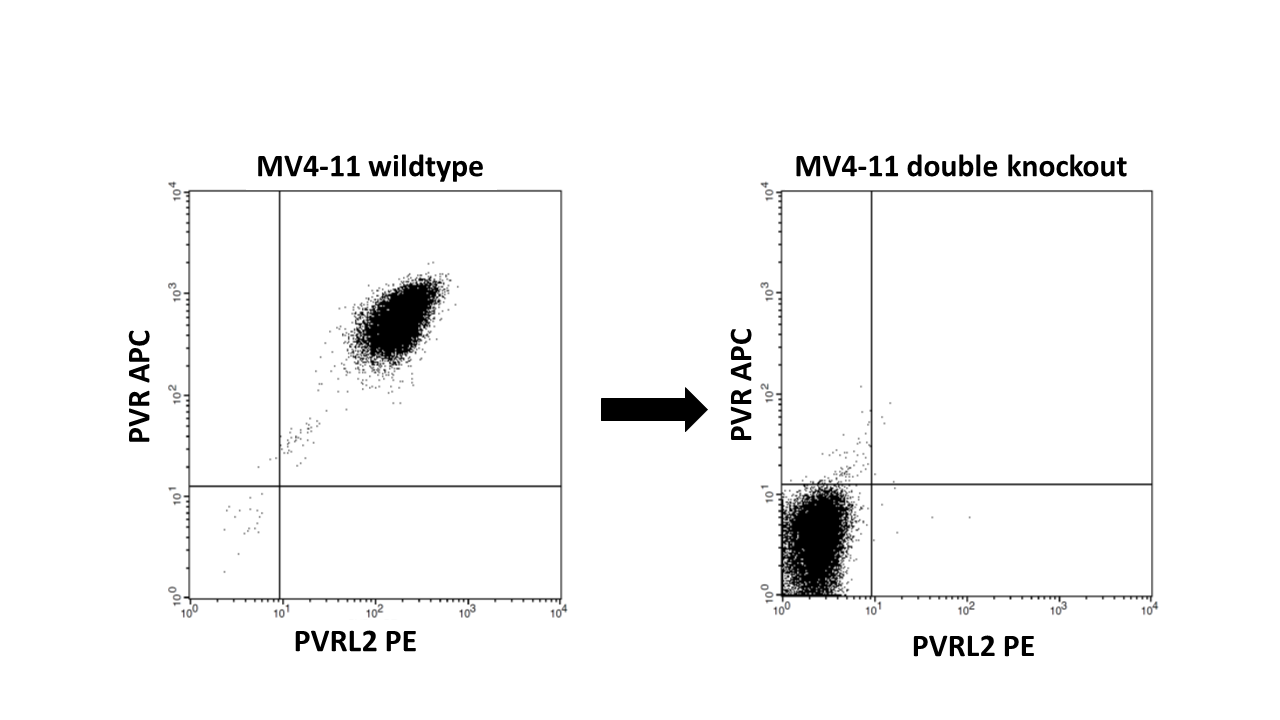
**

**Supplemental Figure S7. FACS analysis for PVR and PVRL2 protein expression of CRISPR/Cas9-generated PVR and PVRL2 double knockout cells.** Protein deficiency of PVR and PVRL2 on the CRISPR/Cas9-generated double knockout cells was verified using flow cytometry. Left plot: MV4-11 wildtype; right plot: MV4-11 PVR and PVRL2 knockout.
