# Supplementary material for: Immune checkpoints PVR and PVRL2 are prognostic markers in AML and their blockade represents a new therapeutic option
Source: Oncogene. 2018 May 31;37(39):5269–80. doi: 10.1038/s41388-018-0288-y (PMC6160395; doi:10.1038/s41388-018-0288-y)
Supplement: Supplementary file 9 — Supplemental Figure S8 [file 41388_2018_288_MOESM9_ESM.docx]

Stamm *et al.,* “**Immune Checkpoints PVR and PVRL2 are Prognostic Markers in AML and Their Blockade Represents a New Therapeutic Option**”


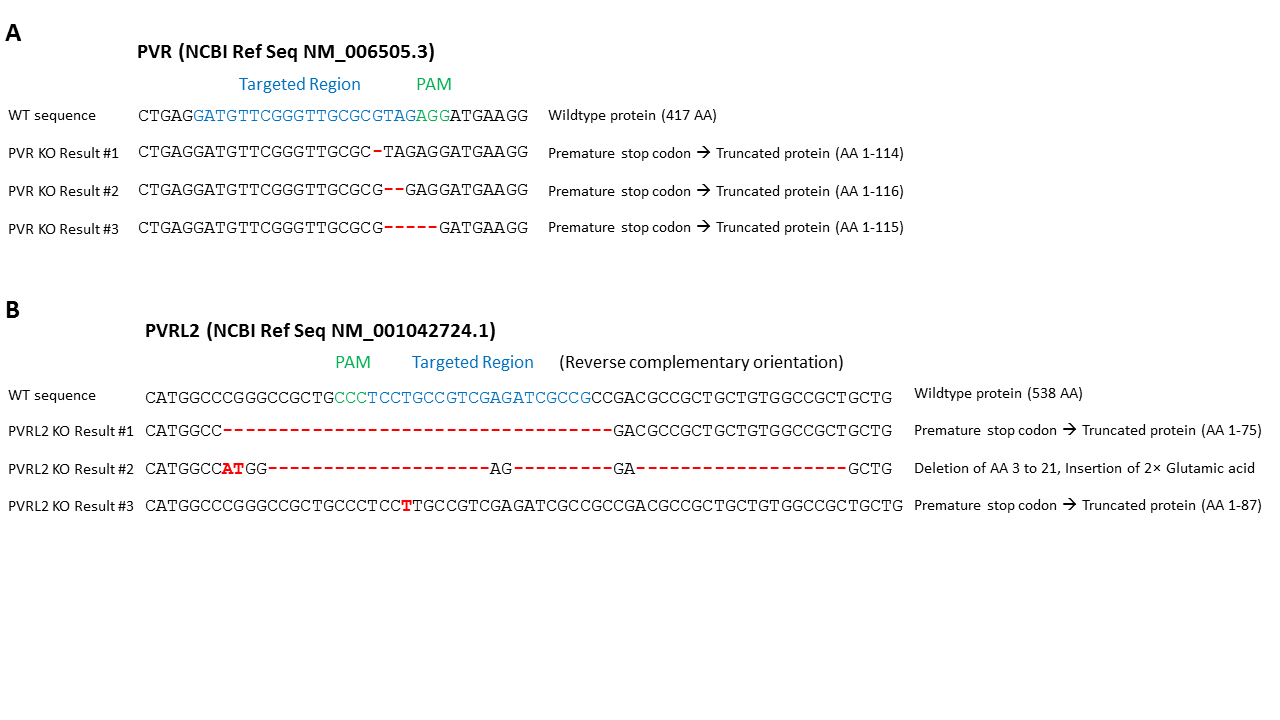


**Supplemental Figure S8. Genomic analysis of CRISPR/Cas9-mediated PVR and PVRL2 double knockout cells.** To validate the CRISPR/Cas9-mediated knockout of PVR and PVRL2 in MV4-11 cells on the genomic level, the corresponding gene sections of several single cells were analyzed by subcloning and sequencing. The genomic alterations for three different knockout clones including the impact on the protein sequence are presented for PVR (A) and PVRL2 (B), respectively. The wildtype sequence with the target sites in blue and the PAM sequence in green is shown at the top. For PVRL2, the targeted region and PAM sequence are in reverse complementary orientation as the PVRL2 guide RNA recognized the antiparallel DNA strand. Deletions within the subclones are shown as red dashes and insertions are shown in red. WT = wildtype, KO = double knockout, PAM = protospacer adjacent motif, AA = amino acid.
