# Supplementary material for: Immune checkpoints PVR and PVRL2 are prognostic markers in AML and their blockade represents a new therapeutic option
Source: Oncogene. 2018 May 31;37(39):5269–80. doi: 10.1038/s41388-018-0288-y (PMC6160395; doi:10.1038/s41388-018-0288-y)
Supplement: Supplementary file 10 — Supplemental Figure S9 [file 41388_2018_288_MOESM10_ESM.docx]

Stamm *et al.,* “**Immune Checkpoints PVR and PVRL2 are Prognostic Markers in AML and Their Blockade Represents a New Therapeutic Option**”


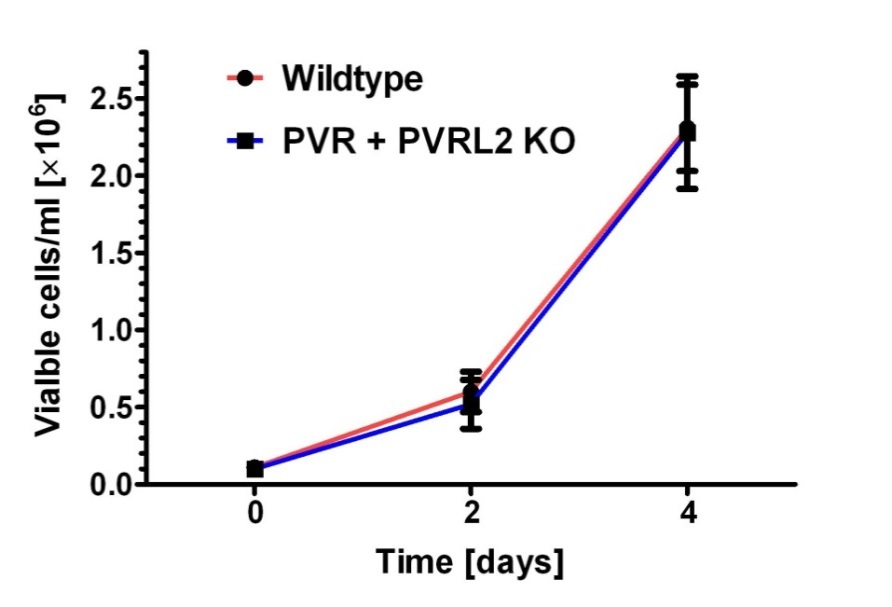


**Supplemental Figure S9. Proliferation capacity of CRISPR/Cas9-generated knockout cells.** The growth rate of MV4-11 PVR and PVRL2 double knockout cells was compared to the proliferation capacity of MV4-11 wildtype cells. Cell counts were measured in technical triplicates on day 2 and 4 using the Vi-Cell™ XR automatic cell counter (Beckman Coulter); n=3.
