# Supplementary material for: Immune checkpoints PVR and PVRL2 are prognostic markers in AML and their blockade represents a new therapeutic option
Source: Oncogene. 2018 May 31;37(39):5269–80. doi: 10.1038/s41388-018-0288-y (PMC6160395; doi:10.1038/s41388-018-0288-y)
Supplement: Supplementary file 11 — Supplemental Figure S10 [file 41388_2018_288_MOESM11_ESM.docx]

Stamm *et al.,* “**Immune Checkpoints PVR and PVRL2 are Prognostic Markers in AML and Their Blockade Represents a New Therapeutic Option**”


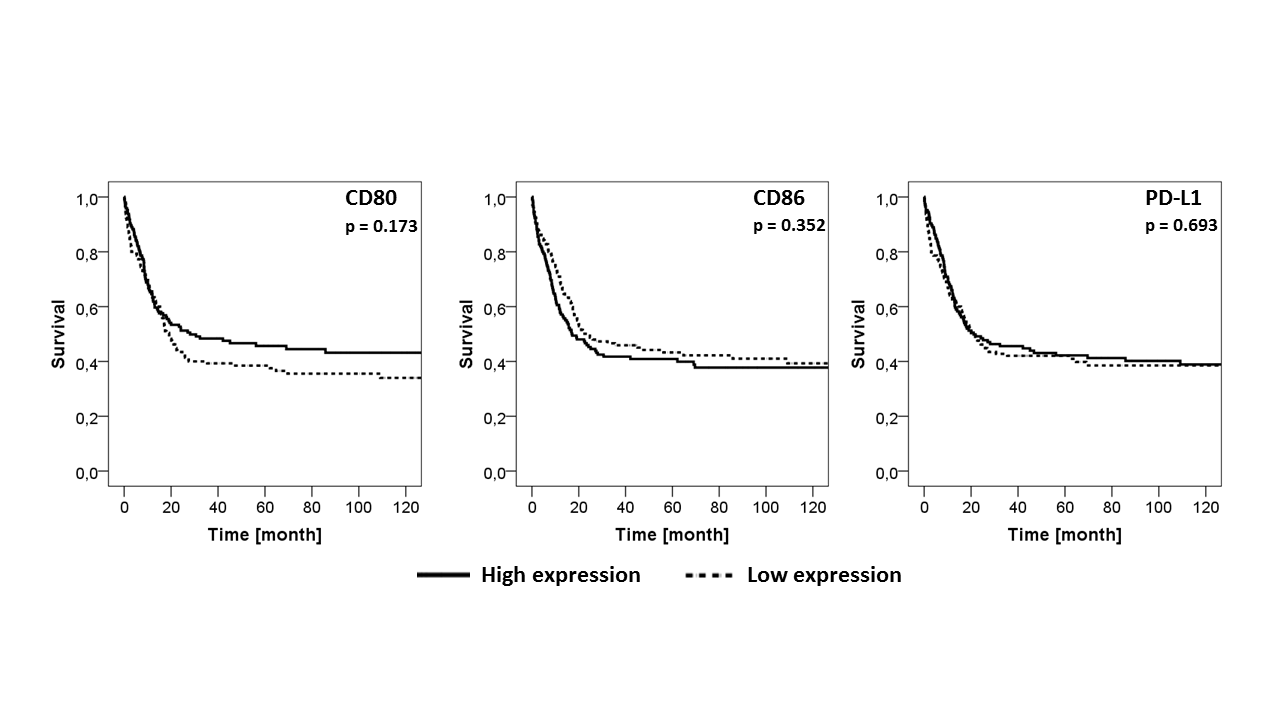


**Supplemental Figure S10. Kaplan-Meier survival analysis of AML patients enclosed in cohort B (n=290).** No significant association between the expression of CTLA-4 ligands *CD80* and *CD86* as well as PD-1 ligand *PD-L1* and the overall survival of AML patients in cohort B was found. For PD-L2 no expression was detectable in this dataset.(1)

Reference:

1. Verhaak RGW, Wouters BJ, Erpelinck CAJ, Abbas S, Beverloo HB, Lugthart S, et al. Prediction of molecular subtypes in acute myeloid leukemia based on gene expression profiling. Haematologica. 2009 Jan 1;94(1):131–4.
