# Supplementary material for: Immune checkpoints PVR and PVRL2 are prognostic markers in AML and their blockade represents a new therapeutic option
Source: Oncogene. 2018 May 31;37(39):5269–80. doi: 10.1038/s41388-018-0288-y (PMC6160395; doi:10.1038/s41388-018-0288-y)
Supplement: Supplementary file 12 — Supplemental Table S1 [file 41388_2018_288_MOESM12_ESM.docx]

Stamm *et al.,* “**Immune Checkpoints PVR and PVRL2 are Prognostic Markers in AML and Their Blockade Represents a New Therapeutic Option**”

**Supplemental Table S1. Patient characteristics of cohort A and cohort B.**

| **Patient characteristics** | **Cohort A (n=139)** | **Cohort B (n=290)** |
| --- | --- | --- |
| Median age in years (range) | 47 (19 - 61) | 44 (15 -60) |
| Sex (male) | 53% | 44% |
| Unfavorable karyotype | 31% | 35% |
| FLT3 mutated | 21% | 41% |
