# Supplementary material for: Immune checkpoints PVR and PVRL2 are prognostic markers in AML and their blockade represents a new therapeutic option
Source: Oncogene. 2018 May 31;37(39):5269–80. doi: 10.1038/s41388-018-0288-y (PMC6160395; doi:10.1038/s41388-018-0288-y)
Supplement: Supplementary file 13 — Supplemental Table S2 [file 41388_2018_288_MOESM13_ESM.docx]

Stamm *et al.,* “**Immune Checkpoints PVR and PVRL2 are Prognostic Markers in AML and Their Blockade Represents a New Therapeutic Option**”

**Supplemental Table S2. Distribution of low vs. high expressors of PVR and PVRL2 for AML FAB subtypes in patient cohort B.**

| **FAB subtype*** | **PVR (%)** | | | **PVRL2 (%)** | | |
| --- | --- | --- | --- | --- | --- | --- |
|  | low | high | Fisher’s exact test | low | high | Fisher’s exact test |
| M1 (n=67) | 72 | 28 | p=0.01 | 48 | 52 | p=0.114 |
| M2 (n=64) | 48 | 52 |  | 53 | 47 |  |
| M4 (n=59) | 49 | 51 |  | 64 | 36 |  |
| M5 (n=62) | 47 | 53 |  | 44 | 56 |  |

***** Due to the low number of 20 cases in total, FAB subtypes M0, M3, M6 and M7 were excluded from the subgroup analysis.
