# Supplementary material for: Immune checkpoints PVR and PVRL2 are prognostic markers in AML and their blockade represents a new therapeutic option
Source: Oncogene. 2018 May 31;37(39):5269–80. doi: 10.1038/s41388-018-0288-y (PMC6160395; doi:10.1038/s41388-018-0288-y)
Supplement: Supplementary file 14 — Supplemental Table S3 [file 41388_2018_288_MOESM14_ESM.docx]

Stamm *et al.,* “**Immune Checkpoints PVR and PVRL2 are Prognostic Markers in AML and Their Blockade Represents a New Therapeutic Option**”

**Supplemental Table S3. Primer sequences.**

| **Primer name** | **Sequence (5'->3')** | **Used for** |
| --- | --- | --- |
| PVR forw subclone | GCTACCTACAGGTGCCCAAC | Amplification of CRISPR/Cas9 targeted region |
| PVR rev subclone | CTCCAGTGAGCTGGACCTTC |  |
| PVRL2 forw subclone | CCCCACAGGCACCTACTAAA |  |
| PVRL2 rev subclone | ACCCATCTTAGGGTGGAAGG |  |
| pJet1.2 forward sequencing primer | CGACTCACTATAGGGAGAGCGGC | Sequencing of subcloned amplicons |
| PVR forw 693 | AGCAGGAGCGTGGATATCTG | RT-qPCR |
| PVR rev 899 | GACTGTGCCAGACAGGAACC |  |
| PVRL2 forw 787 | GAGGACGAGGGCAACTACAC |  |
| PVRL2 rev 989 | AGGGATGAGAGCCAGGAGAT |  |
| GAPDH forw 822 | GTCAGTGGTGGACCTGACCT |  |
| GAPDH rev 1066 | TGCTGTAGCCAAATTCGTTG |  |
